# Epigenetic maps of pearl millet reveal a prominent role for CHH methylation in regulating tissue-specific gene expression

**DOI:** 10.1007/s42994-025-00243-2

**Published:** 2025-08-26

**Authors:** Lin Luo, Qi Qu, Mengxue Cao, Yihui Zhang, Yuanchang Sun, Fei Mao, Jiaming Chen, Yilin Zhu, Yaorou Yang, HuachengLiu, Chunxiao Li, Dongmei Lin, Guodong Lu, Zhanxi Lin, Fangjie Zhu, Jiajing Xiao

**Affiliations:** 1 College of Life Science, National Engineering Research Center of JUNCAO, Fujian Provincial Key Laboratory of Haixia Applied Plant Systems Biology, Haixia Institute of Science and Technology, Fujian Agriculture and Forestry University, Fuzhou, 350002 China; 2 Juncao Science and Ecology College, Fujian Agriculture and Forestry University, Fuzhou, 350002 China; 3 Life Sciences Institute, Zhejiang University, Hangzhou, 310058 China

**Keywords:** Chromatin accessibility, DNA methylation, Histone modification, Tissue-specific transcriptional regulation, WRKY, CHH methylation

## Abstract

Pearl millet (*Pennisetum glaucum*) is a major staple food in arid and semi-arid regions of sub-Saharan Africa, India, and South Asia. However, how epigenetic mechanisms regulate tissue-specific gene expression in this crop remains poorly understood. In this study, we profiled multiple epigenetic features in the young panicles and roots of pearl millet using RNA-seq, ATAC-seq, whole-genome bisulfite sequencing, and ChIP-seq (H3K4me3 and H3K36me3). We identified thousands of genes that were differentially expressed between these two tissues. Root-specific genes were enriched for plant hormone signaling, oxidative phosphorylation, and stress responses. Analysis of chromatin accessibility revealed that root-specific accessible chromatin regions (ACRs) were enriched in binding motifs for stress-responsive transcription factors (e.g., NAC, WRKY), whereas ACRs in young panicles were enriched in motifs for developmental regulators (e.g., AP2/ERF). DNA methylation profiling revealed 25,141 tissue-specific differentially methylated regions, with CHH methylation—rather than CG or CHG methylation—showing the strongest tissue specificity. Promoters of root-specific genes had higher levels of CHH methylation compared to those of young panicle–specific genes, suggesting that the roles of CHH methylation in regulating transcription might be tissue dependent. Notably, promoter-associated H3K4me3 marked panicle-specific genes, whereas root-specific expression was primarily linked to chromatin accessibility, suggesting a transcription factor–mediated regulatory mechanism. Together, our findings highlight the distinct epigenetic frameworks governing tissue-specific gene expression in pearl millet and provide valuable insights for advancing the genetic improvement of this crop.

**Supplementary Information:**

The online version contains supplementary material available at 10.1007/s42994-025-00243-2.

## Introduction

Global climate change has intensified the threats of high temperatures and drought, posing a significant risk to crop growth and yields (Challinor et al. 2014; Food Security and Food Production Systems 2014; Jacott and Boden 2020; Sun et al. 2022a). Pearl millet [*Pennisetum glaucum* (L.) R.Br., syn. *Cenchrus americanus* L. Morrone], a C4 crop in the Poaceae family, has gained significant recognition for its exceptional ability to tolerate a wide range of environmental stresses, as well as its notable nutritional value (Satyavathi et al. 2021). Cultivated in over 30 countries, pearl millet is the sixth most widely planted cereal crop globally, playing a vital role in ensuring food security and alleviating hunger. Pearl millet also exhibits advantageous traits, including a short life cycle, rapid growth rate, high photosynthetic efficiency, and adaptability to low irrigation and poor soil fertility conditions (Singh and Nara 2023).

Recent advancements in high-throughput sequencing technologies have facilitated the high-quality assembly of the pearl millet genome (Ramu et al. 2023; Varshney et al. 2017). High-quality *de novo* genome assemblies for 10 pearl millet accessions were recently generated to construct a graph-based pan-genome, encompassing 424,085 annotated structural variants (SVs) (Yan et al. 2023). These SVs have been leveraged in association analyses, providing valuable insights into the genetic basis of heat tolerance in pearl millet. In parallel, comprehensive transcriptome profiling across tissues and developmental stages has enabled the identification of housekeeping genes and tissue-specific expression patterns, enhancing our understanding of gene function and transcriptional dynamics throughout the pearl millet life cycle (Luo et al. 2024). A spatiotemporal transcriptome of pearl millet has revealed key genes and regulatory modules controlling stalk development, offering a molecular framework for dissecting growth dynamics and developmental regulation in this climate-resilient crop (Mao et al. 2024). Furthermore, online databases have been developed to host genomic sequences, gene annotations, and transcriptomic datasets for pearl millet, thereby facilitating data accessibility and reuse for downstream research and breeding applications (Sun et al. 2023). Genes and quantitative trait loci have been identified to elucidate the genetic basis of stress tolerance, leading to the development of improved cultivars with enhanced yield potential and nutritional quality (Khan et al. 2020; Yadav et al. 2021; Yan et al. 2023). However, the regulatory elements embedded within the extensive non-coding regions of the pearl millet genome remain largely uncharacterized, limiting our understanding of the molecular mechanisms underlying gene regulation in this important crop.

Genome-wide analysis of the WRKY transcription factor (TF) family in pearl millet revealed that several WRKY genes are differentially expressed under dehydration or salinity stress compared to normal conditions, highlighting their potential roles in the transcriptional regulation of abiotic stress responses (Chanwala et al. 2020). Furthermore, functional characterization of *CaWRKY52* (*PgWRKY52*) demonstrated that stress-responsive *cis*-regulatory elements within the promoters of its target genes mediate abscisic acid–methyl jasmonate-dependent transcriptional regulation, thereby enhancing salt stress tolerance (Chanwala et al. 2025). These findings emphasize the critical roles of non-coding regulatory sequences in modulating stress-responsive gene expression and underscore the need for systematic identification and functional annotation of the non-coding regions of the pearl millet genome.

The construction of epigenomic maps offers critical insights into regulatory landscapes, particularly within the non-coding regions of genes that harbor enhancers, silencers, and other *cis*-regulatory elements (Li et al. 2025; Marand et al. 2023; Zhang et al. 2021). Recent epigenomic studies in various crop species have shown that the spatiotemporal regulation of gene expression is tightly controlled by tissue-specific epigenetic features, including chromatin accessibility, histone modifications such as H3K4me3 and H3K36me3, and context-dependent DNA methylation (Liu et al. 2023; Zhang and Zhu 2025; Zhong et al. 2013). In addition, DNA methylation regulates gene transcription by influencing chromatin structure and the recognition of regulatory elements by TFs (Bender 2004; He et al. 2025). We previously established the first epigenomic map of pearl millet using leaf tissue, which identified leaf-specific accessible chromatin regions (ACRs) and demonstrated that H3K4me3 is associated with the activation of C_4_ photosynthetic genes, such as *PEPCK* (Luo et al. 2025). Despite these advances, a comprehensive understanding of how epigenetic features vary across tissues, such as root (RO) and young panicle (YP) tissue, and how they contribute to organ-specific gene expression is lacking. It remains unclear to what extent chromatin accessibility, histone modifications, and DNA methylation determine the transcriptional programs during developmental transitions and stress responses. Addressing this gap is crucial for fully leveraging epigenetic information in functional genomics and stress biology studies, as well as for trait improvement in pearl millet.

Here, we present an integrative epigenomic atlas for RO and YP tissues of pearl millet, combining information about ACRs, H3K4me3, H3K36me3, DNA methylation (CG, CHG, and CHH), and gene expression. Through comparative analysis with previously generated epigenome data for leaf tissue, we systematically characterized the tissue-specific transcriptional activity and related epigenetic features in this crop. Our results emphasize the dominant roles of ACR and CHH methylation in root-specific gene expression and reveal how transposable elements (TEs) and their epigenetic features shape the transcriptional landscape of pearl millet.

## Results

### RO and YP tissue exhibit distinct chromatin accessibility and epigenetic landscapes

**Fig. 1:**
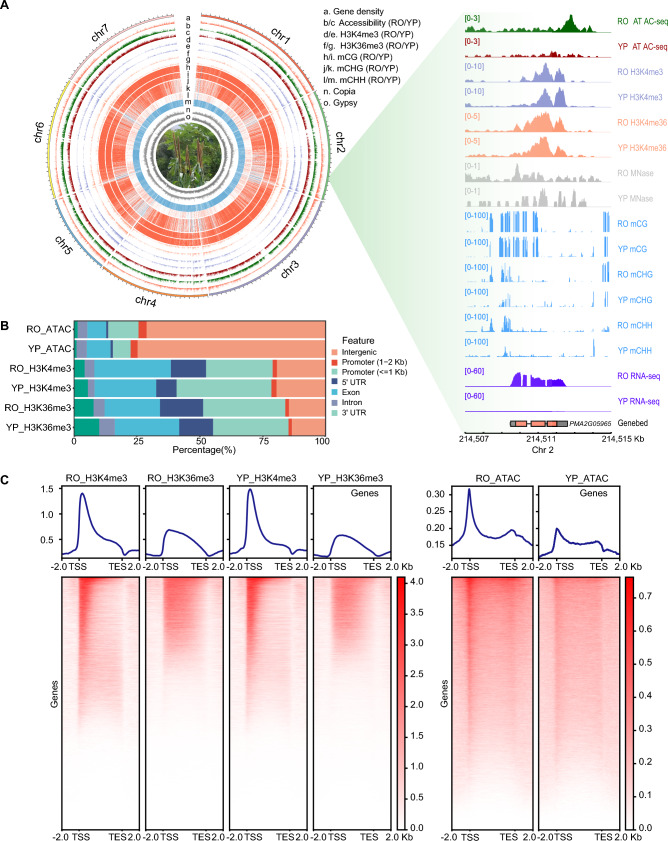
Genome-wide distribution of chromatin accessibility, histone marks, and DNA methylation in root (RO) and young panicle (YP) tissues of pearl millet. **A** Genome-wide distribution of gene density (a), chromatin accessibility (b for RO and c for YP), H3K4me3 (d for RO and e for YP), H3K36me3 (f for RO and g for YP), CG methylation (h for RO and i for YP), CHG methylation (j for RO and k for YP), CHH methylation (l for RO and m for YP), and transposable elements (TEs, n for Copia and o for Gypsy). **B** Genome Browser view of the *PMA5G06576* locus showing chromatin accessibility, histone modifications, DNA methylation, nucleosome occupancy, and gene expression in RO and YP tissues. **C** Genomic distribution of chromatin features, including ATAC-seq peaks, H3K4me3, and H3K36me3, in RO and YP. Heatmaps and aggregated signal profiles showing the distribution of histone modifications (H3K4me3 and H3K36me3) and chromatin accessibility (ATAC-seq) around gene bodies (± 2 kb from the transcription start site [TSS] and transcription end site [TES]) in RO and YP tissues of pearl millet

To reveal the epigenetic features of RO and YP tissue in pearl millet, we performed a comprehensive multi-omics study incorporating assays for transposase-accessible chromatin using sequencing (ATAC-seq), whole-genome bisulfite sequencing (WGBS), chromatin immunoprecipitation sequencing (ChIP-seq) for two histone modifications (H3K4me3 and H3K36me3), and total mRNA sequencing (RNA-seq). The resulting epigenetic and transcriptomic datasets comprise 72.00 Gb of WGBS data, 22.92 Gb of ATAC-seq data, 28.90 Gb of ChIP-seq data, and 29.74 Gb of RNA-seq data, providing a rich resource for analyzing chromatin accessibility, DNA methylation, histone modifications, and gene expression patterns in both tissues (Fig. 1A). Our findings offer important insights into the tissue-specific regulation of gene expression. For instance, the *NPR1*-like gene *PMA5G02665*, displaying clear RO-specific expression, is associated with RO-specific accessible signals (Fig. 1B). Similarly, *JAZ1* (*PMA5G00676*), which is specifically expressed in RO tissue, is associated with higher CHH methylation level in RO (Fig. S1).

A comprehensive assessment of the quality of the peak-calling results indicated that the ATAC-seq datasets were of moderate to high quality, making them suitable for downstream analysis (Fig. S2). Moreover, the two biological replicates of ATAC-seq data for each tissue are in good agreement (Pearson’s *r* > 0.9), confirming the high quality and reproducibility of the data (Fig. S3). Using the merged datasets, we identified 128,110 and 128,025 ACRs in RO and YP, respectively. A substantial proportion of these ACRs—71.07% in RO and 74.65% in YP—were located in intergenic regions, which is consistent with previous DNase-seq results in leaf tissue (Luo et al. 2025) (Fig. 1C), suggesting the widespread distribution of distal regulatory *cis-*elements in pearl millet. Using established methods, we identified 34,610 H3K4me3 and 23,123 H3K36me3 peaks in RO and 37,929 H3K4me3 and 21,003 H3K36me3 peaks in YP. Analysis of the distribution patterns of H3K4me3, H3K36me3, and ACRs relative to gene bodies revealed distinct chromatin signatures, underscoring their roles in regulating transcriptional activity (Fig. 1C). Consistent with epigenomic features previously observed in new leaf (NL) tissue, H3K4me3 signals were predominantly enriched near the transcription start sites (TSSs) of genes, consistent with the role of H3K4me3 in marking active promoters and the 5′ ends of transcribed regions (Fig. S4).

Furthermore, the ACRs exhibited a distribution pattern consistent with gene density. The gene-rich regions were highly enriched for H3K4me3 and H3K36me3, reflecting active transcriptional regulation. By contrast, gene-depleted regions were associated with high levels of CG and CHG DNA methylation, particularly within TE-enriched regions, reinforcing the roles of DNA methylation in TE silencing and genome stability. Genomic regions characterized by low CG and CHG methylation corresponded to transcriptionally active sites, as indicated by the strong ATAC-seq, H3K4me3, and H3K36me3 signals, demonstrating the tight coordination between DNA methylation and other epigenetic features in regulating gene expression.

### Transcriptomic landscape of RO and YP reveals tissue-specific expression programs in pearl millet

**Fig. 2:**
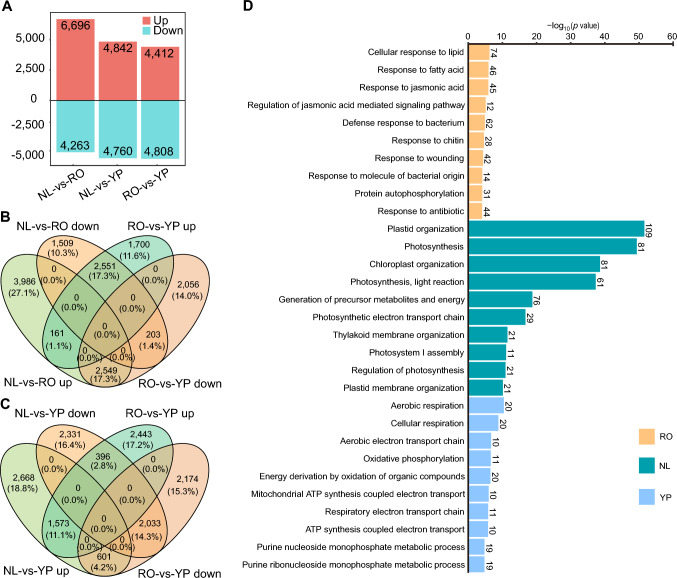
Tissue-specific gene expression and functional enrichment analysis. **A** Bar plot showing the number of differentially expressed genes across the three comparisons: new leaf (NL) vs. RO, NL vs. YP, and RO vs. YP. The bars represent the number of DEGs, and the colors of the bars indicate upregulated DEGs (red) or downregulated (blue) DEGs. **B, C** Venn diagrams displaying the overlap of upregulated DEGs and downregulated DEGs identified in RO (**B**) and YP (**C**), respectively, when each tissue is compared to the remaining two tissues. **D** Top 10 enriched biological processes for genes expressed in specific tissues in RO (orange), NL (green), and YP (blue). The *y*-axis represents the − log_10_(*p*-value) of significantly enriched biological processes, while the *x*-axis lists the GO terms

To investigate tissue-specific transcriptional regulation in pearl millet, we performed a comparative analysis of differentially expressed genes (DEGs) across YP, RO, and NL tissues by integrating the current data with our recently published transcriptomic dataset (Luo et al. 2025). A total of 4263–6696 DEGs were identified across these tissues (Fig. 2A), among which 1405–2877 genes exhibited tissue-specific expression patterns (Fig. 2B, C, and Fig. S5). To gain insights into the biological functions of these genes, we conducted Gene Ontology (GO) enrichment analysis for each tissue (Fig. 2D). GO enrichment analysis of the 2,551 genes specifically upregulated in RO compared to YP and NL revealed their associations with key biological processes such as “cellular response to lipids”, “jasmonic acid (JA) signaling”, and “defense response to bacterium”. These functions highlight the critical roles of root-specific genes in stress adaptation, plant hormone signaling, and environmental responses. Notably, 228 TF genes were highly expressed specifically in RO, including 33 NAC TF and 29 WRKY TF family members (Fig. S6), which are known to regulate abiotic stress responses, root development, and immune signaling pathways.

In NL, 2877 genes showed tissue-specific expression; significantly enriched GO terms included “photosynthesis”, “chloroplast organization”, and “photosynthesis, light reaction” (Fig. 2D and Fig. S7). These findings are consistent with the roles of young leaves as the primary sites of light energy capture and chloroplast activity, suggesting that genes involved in chloroplast biogenesis, light harvesting, and carbon fixation are transcriptionally activated in NL. GO enrichment analysis of the 2,033 genes specifically expressed in YP compared with NL and RO revealed their involvement in “cellular respiration” and “oxidative phosphorylation”, reflecting the high energy demands of floral and reproductive development. These pathways are essential for supporting cell proliferation, organ differentiation, and nutrient transport during the formation of reproductive structures. Overall, the distinct expression profiles across tissues underscore the specialized transcriptional programs driving RO function, photosynthetic activity in leaves, and metabolic energy support for panicle development. This tissue-specific transcriptional regulation forms the molecular foundation for the functional adaptation of pearl millet.

### Landscape of ACRs differs markedly between RO and YP

**Fig. 3:**
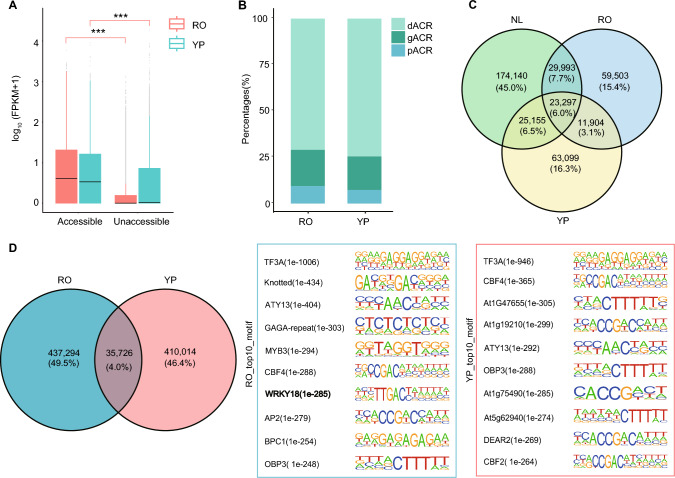
Comparative analysis of chromatin accessibility and transcription factor–binding motifs across RO and YP in pearl millet. **A** Boxplot comparing gene expression levels [log_10_-transformed (FPKM + 1)] between accessible and inaccessible chromatin regions in RO and YP. The center line represents the median; the box edges indicate the 25th and 75th percentiles (interquartile range, IQR); whiskers extend to values within 1.5 × IQR from the lower and upper quartiles. Asterisks indicate significant differences (***, *P*-value < 0.001, Student’s *t*-test). **B** Stacked bar plot showing the proportions of distal ACRs (dACR), genic ACRs (gACR), and promoter ACRs (pACR) in RO and YP. **C** Venn diagram illustrating the overlap of accessible chromatin regions (ACRs) among RO, YP, and NL. **D** Venn diagram comparing the number of TF footprints in RO and YP. The blue and red boxes at right show the top 10 most significantly enriched transcription factor–binding motifs and their corresponding *P*-values in RO and YP, respectively

Chromatin accessibility is a key determinant of gene expression, as it enables TFs and other regulatory proteins to access DNA and regulate transcription (Klemm et al. 2019). In pearl millet, we identified 19,252 genes (37.58%) in RO and 13,424 genes (26.21%) in YP with ACRs in their promoter regions. These genes with promoter-associated ACRs showed significantly higher expression levels than genes lacking ACRs in their promoters (Fig. 3A), which is consistent with previous findings (Zhao et al. 2020). In addition to promoter accessibility, a substantial proportion of ACRs was located in the distal regions of genes, suggesting the involvement of long-range, enhancer-like regulatory elements. Specifically, 90,958 ACRs (71.00%) in RO and 95,480 ACRs (74.58%) in YP were located more than 2 kb away from the nearest annotated gene (Fig. 3B). This distribution pattern mimics the pattern previously observed in leaves, indicating that distal regulatory regions, such as enhancers or tissue-specific TF binding sites, play a dominant role in shaping transcriptional activity in pearl millet.

To investigate tissue-specific regulatory elements in pearl millet, we compared ACRs identified in RO and YP using ATAC-seq with those detected in NL (Fig. 3C). Among the 255,629 ACRs identified in NL, the vast majority were not shared with RO or YP, indicating distinct chromatin accessibility landscapes in NL. Only 23,297 common ACRs (6%) were detected across all three tissues (NL, RO, and YP), suggesting the widespread presence of tissue-specific distal regulatory elements.

To assess transcription factor–binding sites (TFBSs), we performed HINT-ATAC, a footprinting method that corrects for Tn5 transposase cleavage bias (Li et al. 2019). This analysis identified 514,245 footprints in RO and 488,786 footprints in YP, far exceeding the number of footprints previously identified in NL. Despite the large number of footprints in each tissue, only 35,726 footprints were shared between RO and YP, representing a small fraction of the total TFBSs, suggesting that TF binding is likely highly dynamic and tissue specific (Fig. 3D). Motif enrichment analysis of the footprints revealed 47 NAC and 30 WRKY family motifs that were significantly enriched in RO, supporting their involvement in root-specific transcriptional regulation. This is also consistent with the observation that WRKY activity was positively correlated with chromatin accessibility in Arabidopsis (*Arabidopsis thaliana*) only in RO tissue (Wen et al. 2023). Among these, TFs such as WRKY55 (PMA1G03753, PMA5G01779), WRKY40 (PMA2G00644), and WRKY50 (PMA1G05754, PMA1G06480, PMA6G03296) emerged as potential key regulators of root development and stress responses. By contrast, AP2 family motifs were predominantly enriched in YP footprints, indicating their central roles in panicle-specific gene regulation and floral development.

To examine tissue-specific differences in chromatin accessibility, we identified significant differentially accessible regions (DARs) between RO and YP. Compared to YP, 5269 gained and 980 lost DARs were detected in RO. In RO, a relatively large proportion of ACRs were located near gene-associated regions (201; ~ 59.31%) and putative enhancers (2068; ~ 40.69%). By contrast, ACRs identified in YP appeared to be more enriched in distal enhancer-like regions (694; ~ 70.81%) (Fig. S8). The gained DARs were associated with 2976 genes, including 1470 DEGs (1017 upregulated DEGs and 453 downregulated DEGs in RO compared to YP), indicating that changes in chromatin accessibility likely contribute to the tissue-specific activation of gene expression programs. Functional enrichment analysis revealed that genes linked to gained DARs in RO were significantly enriched in the JA signaling pathway (Fig. S9), suggesting that JA-mediated gene regulation is at least partially controlled by chromatin accessibility in RO. In addition, KEGG pathway enrichment analysis indicated that these genes were overrepresented in pathways such as “Plant hormone signal transduction” and “MAPK signaling pathway”, both of which are central to stress responses and environmental signal integration.

### Coordinated regulation of gene expression by histone modifications and ACRs in RO and YP

**Fig. 4:**
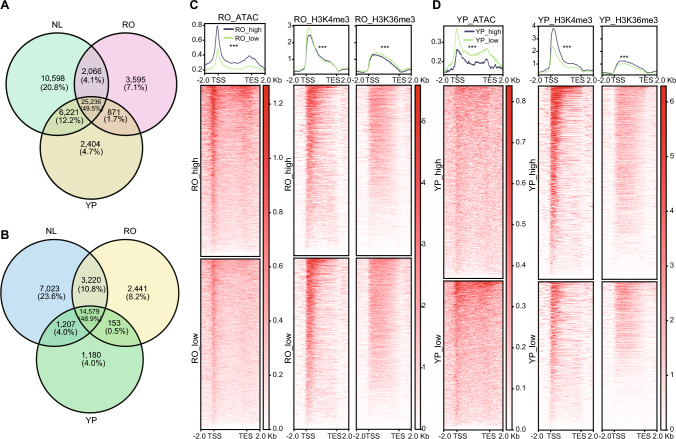
Comparative analysis of histone modifications, chromatin accessibility, and differential gene expression across tissues. **A, B** Venn diagrams showing the distribution of H3K4me3 (**A**) and H3K36me3 (**B**) histone modifications across RO, YP, and NL tissues in pearl millet. **C, D** Aggregated profiles and heatmaps showing the distribution of ATAC-seq, H3K4me3, and H3K36me3 signals at transcription start sites (TSSs) and transcription end sites (TESs) for genes with differential expression in RO (**C**) and YP (**D**). The average signal intensities of ATAC-seq, H3K4me3, and H3K36me3 shown at the top of panels C and D are plotted across gene body regions (from TSS ± 2 kb to TES ± 2 kb) for genes with high or low expression levels in RO and YP. Statistical significance between the high- and low-expression groups was evaluated using a two-tailed Student’s *t*-test, with asterisks indicating significance levels (***, *p* < 0.001)

Histone modifications, particularly H3K4me3 and H3K36me3, are well-known epigenetic marks associated with transcriptional activation. Across all three tissues, 25,236 H3K4me3-marked regions (49.5%) and 14,579 H3K36me3-marked regions (48.9%) were shared, indicating that a core set of constitutively expressed genes is present across multiple tissues (Fig. 4A–B). This stands in stark contrast to the minimal sharing of ACRs across tissues (4.0%), suggesting that ACRs contribute relatively weakly to the expression of the core set of genes. However, when we focused on tissue-specific gene expression, distinct epigenetic patterns emerged. In RO, the specifically expressed genes exhibited higher chromatin accessibility (ACRs), which could enable the binding of root-specific TFs (Fig. 4C). Counterintuitively, histone modifications contributed little to root-specific gene regulation. Among the 2,551 root-specific genes, only 688 were marked by root-specific H3K36me3 and 346 by root-specific H3K4me3. Notably, 632 of these genes were marked by YP-specific H3K4me3 instead. These findings further support the idea that gene expression in RO is primarily associated with chromatin openness rather than histone modifications. By contrast, highly expressed genes in YP exhibited strong H3K4me3 and ACR enrichment in their promoter regions, along with broad H3K36me3 distribution across gene bodies, highlighting the notion that chromatin accessibility and histone modifications cooperatively promote transcription during reproductive development (Fig. 4D).

### RO exhibits globally elevated CHH methylation compared to YP

**Fig. 5:**
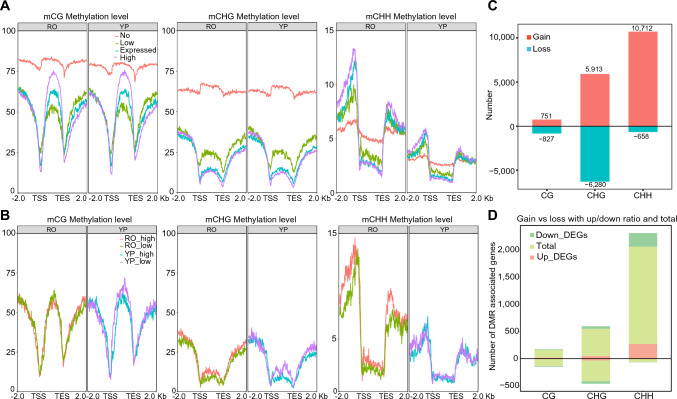
DNA methylation landscape across different genomic features and tissues in pearl millet. **A** DNA methylation levels (CG, CHG, and CHH contexts) across promoter regions, gene bodies, and downstream regions in RO and YP. Methylation patterns are categorized by gene expression levels (not expressed [No, FPKM = 0], expressed at low levels [Low, 0 < FPKM < 1], expressed at moderate levels [Expressed, 1 ≤ FPKM < 100], and expressed at high levels [High, FPKM ≥ 100]). **B** DNA methylation levels (CG, CHG, and CHH contexts) across promoter regions, gene bodies, and downstream regions for genes expressed in specific tissues. **C** Bar plot displaying the number of differentially methylated regions (DMRs) between RO and YP. The panel shows the number of hypermethylated (red) and hypomethylated (blue) regions across the CG, CHG, and CHH contexts. **D** DMRs that overlap with DEGs between RO and YP

DNA methylation is a key epigenetic modification that regulates gene transcription and transposon silencing (Mattei et al. 2022). Using WGBS, we evaluated the DNA methylation of cytosine residues in RO and YP. While the levels of CG and CHG methylation were comparable between the two tissues, CHH methylation at the promoter regions of genes was markedly higher in RO, suggestive of a distinct regulatory landscape in RO (Fig. S10). To further explore the relationship between methylation and transcription, we examined DNA methylation profiles across genes binned by their expression levels (i.e., not expressed or expressed at low, moderate, and high levels) (Fig. 5A). As expected, high levels of CG and CHG methylation at promoters were associated with transcriptional repression, while CG methylation at gene bodies was positively correlated with gene expression. These observations are consistent with previously reported cytosine methylation patterns (Mattei et al. 2022).

Compared to cytosine methylation, CHH methylation was more dynamic and correlated with active gene transcription, particularly in RO, suggesting that CHH may have a non-canonical regulatory role beyond silencing. Compared to non-expressed genes, significantly higher levels of CHH methylation were observed in the promoters of highly expressed genes (Fig. 5B). We also found significantly higher expression levels of RO-specific genes compared to YP-specific genes (Fig. S11A). CHH methylation levels in the promoters of RO-specific genes were significantly higher than those of YP-specific genes (Fig. S11B). A comparative analysis between RO and YP revealed 17,376 hypermethylated differentially methylated regions (hyper-DMRs) and 7,765 hypomethylated DMRs (hypo-DMRs) in RO (Fig. 5C). We also found a significant correlation between CHH methylation and gene expression in RO, while a weaker association appeared to exist in YP, although this association was not significant (Fig. S12). The majority of these hyper-DMRs were located in intergenic regions, including 69.11% in the CG context, 76.97% in the CHG context, and 71.94% in the CHH context, indicating widespread changes in methylation outside of gene bodies.

To explore the molecular basis of the enriched CHH methylation in RO, we analyzed the expression patterns of genes involved in the RNA-directed DNA methylation (RdDM) pathway, which is the major mechanism driving CHH methylation in plants. Based on canonical RdDM components from Arabidopsis and rice (*Oryza sativa*) (Wang et al. 2021), we identified the homologs of these genes in the pearl millet genome (listed in Table S1) and examined their expression profiles across RO and YP. As shown in the heatmap and volcano plot (Fig. S13, S14), several genes were significantly upregulated in RO, including *CaCMT2* (*PMA1G07308*), *CaNRPD1* (*PMA3G01164*), *CaNRPF1* (*PMA3G02173*), *CaRDR2* (*PMA3G02317*), and *JMJ703* (*PMA3G08188*). These genes are known to participate in small RNA (sRNA) biogenesis, chromatin remodeling, and methylation/demethylation dynamics (Erdmann and Picard 2020). Their elevated expression in RO may contribute to the tissue-specific CHH hypermethylation observed in our dataset.

Notably, 148 to 1,966 hyper-DMRs and 61 to 433 hypo-DMRs were identified in promoter regions, affecting 57–1789 genes (Fig. 5D). Among the affected genes, 272 upregulated and 252 downregulated DEGs overlapped with promoter-localized hyper-CHH DMRs. These findings suggest that changes in CHH methylation at promoters can both activate and repress gene expression in a tissue-specific manner, highlighting the flexible role of this DNA modification.

Because chromatin accessibility, histone modification, and DNA methylation collectively contribute to transcriptional regulation, we further examined DNA methylation patterns within ACRs and histone modification–enriched regions (Fig. S15). In general, CG and CHG methylation levels were reduced in actively transcribed genes, which is consistent with their well-recognized repressive roles. Promoters with ACRs frequently exhibited elevated CHH methylation, suggesting complex interplay between accessibility and CHH methylation. This pattern may reflect a regulatory mechanism whereby CHH methylation fine-tunes promoter activity in accessible chromatin contexts, rather than acting solely as a silencing mark.

### TEs serve as regulatory elements influencing tissue-specific gene expression

**Fig. 6:**
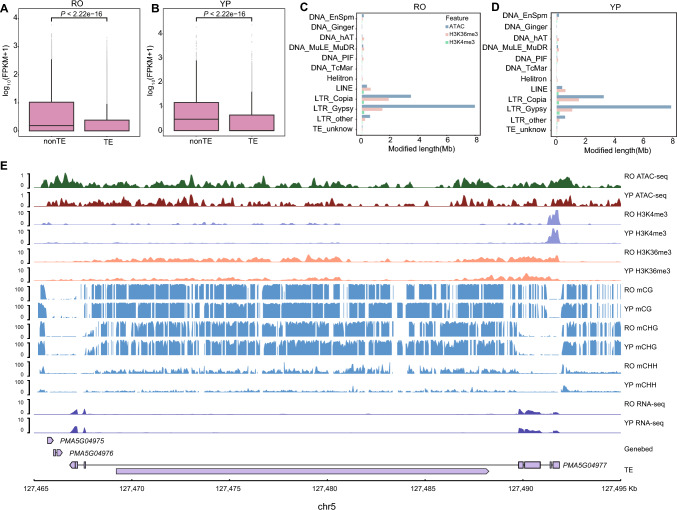
Epigenetic landscape of transposable element (TE)–associated genes in pearl millet. **A**, **B** Boxplots comparing gene expression levels [log_10_-transformed (FPKM + 1)] between TE-associated genes (genes with TEs overlapping their gene body or promoter region) and TE-free genes (genes without any TE association) in RO (**A**) and YP (**B**). The center line represents the median; the box edges indicate the 25th and 75th percentiles (interquartile range, IQR); whiskers extend to values within 1.5 × IQR from the lower and upper quartiles. **C, D** Bar plots displaying the distribution of TE-associated histone modifications across RO (**C**) and YP (**D**). **E** Genome Browser tracks illustrating the distribution of chromatin accessibility (ATAC-seq), histone modifications (H3K4me3 and H3K36me3), and DNA methylation around a representative LTR-associated gene locus

TEs, particularly long terminal repeat (LTR) retrotransposons, have played significant roles in shaping the structure and evolutionary trajectory of the pearl millet genome (Yan et al. 2023). In RO, we identified 40,464 ACRs associated with Gypsy elements, the majority of which (94.01%) are located in intergenic regions, a pattern consistent with their typical distribution. Notably, 1106 Gypsy-associated ATAC peaks were found within gene promoter regions, highlighting their potential involvement in transcriptional regulation (Fig. S16). Genes with TE insertions exhibited significantly reduced expression levels (Fig. 6A, B), suggesting that TEs may contribute to transcriptional silencing. Despite their well-known silencing potential, a substantial proportion of ACRs were found within LTRs: 31.60% in RO and 38.78% in YP (Fig. 6C–D). This observation implies that TEs, particularly LTR elements, may also act as regulatory platforms, influencing chromatin accessibility and in turn gene expression. Consistent with this, we observed strong enrichment of the active histone mark H3K36me3 in LTR regions (Fig. 6E). Specifically, 64.32% of H3K36me3-marked LTR-Gypsy elements and 74.30% of H3K36me3-marked LTR-Copia elements were located within intronic regions, affecting the transcriptional context of 542 and 717 genes in RO, respectively. These findings suggest that LTR insertions may influence not only transcriptional regulation but also alternative splicing, thereby contributing to tissue-specific expression profiles.

Both LTR transposons and TEs exhibited elevated CG and CHG methylation levels compared to genic regions; this methylation may reinforce the transcriptional repressive role of TEs (Fig. S17). Notably, LTR transposons featured a higher level of methylation than DNA elements across both their internal sequences and flanking regions, which is consistent with the commonly observed stronger epigenetic silencing of LTR-type transposons. Furthermore, we observed pronounced differences in methylation between TE-associated genes (genes with TEs overlapping their gene bodies or promoter regions) and TE-free genes (genes not associated with TEs) (Fig. S18). TE-associated genes exhibited higher levels of CG and CHG methylation across their gene bodies, further supporting the presence of robust silencing mechanisms triggered by nearby TE insertions. Altogether, these findings highlight the dual roles of TEs in the pearl millet genome, functioning as both epigenetically silenced elements and regulatory sequences that influence gene expression by affecting chromatin accessibility and histone modification.

## Discussion

In this study, we systematically profiled the epigenetic features of RO and YP tissues of the highly stress-tolerant crop pearl millet. The newly generated data complement our previous epigenetic data from leaf tissue (Luo et al. 2025), offering a comprehensive epigenetic dataset for pearl millet. The availability of epigenetic maps for different tissues allowed us to perform a comparative analysis to identify the determinants of tissue-specific gene expression.

The relationships among DNA methylation, ACRs, TEs, and histone modifications in pearl millet have been poorly understood, although a previous study revealed cooperation between repressive histone marks such as H3K27me3 and non-CG DNA methylation in regulating plant gene expression (Zhou et al. 2016). The respective effect sizes of cytosine methylation and histone modification, and how methylation cooperates with active epigenetic features, such as ACRs and H3K36me3, have been unclear (Du et al. 2015; Li et al. 2021b). Here, we systematically analyzed the effects of DNA methylation on other epigenetic features and on transcriptional activity in different pearl millet tissues (Fig. 5 and Fig. S13).

CG and CHG methylation levels were similar between RO and YP. However, CHH methylation levels were markedly higher in RO than in YP, especially in the promoter regions of root-specific genes (Fig. 5). Consistent with the established roles of DNA methylation in gene regulation (Mattei et al. 2022), higher levels of CG and CHG methylation are associated with lower levels of gene expression, which is also true for CHH methylation. CHH methylation in promoter regions plays a repressive role in gene expression. In *Arabidopsis*, CHH methylation is established and maintained through the RdDM pathway involving DOMAINS REARRANGED METHYLTRANSFERASE 2 (DRM2), as well as a distinct pathway mediated by CHROMOMETHYLASE 2 (CMT2) (Stroud et al. 2014). This has been observed across species such as Arabidopsis (Zhong et al. 2021), rice (Jiang et al. 2025a, b), maize (*Zea mays*) (Liu et al. 2024), wheat (*Triticum aestivum*) (N'Diaye et al. 2020), peanut (*Arachis hypogaea*) (Yu et al. 2025), and tomato (*Solanum lycopersicum*) (Zhong et al. 2013). However, recent studies suggest that CHH methylation may also be associated with transcriptional activation in specific contexts (Fig. S14). For example, in rice endosperm, low CHH methylation levels promote gene expression (Zemach et al. 2010), while in cotton (*Gossypium hirsutum*) and orchard grass (*Dactylis glomerata*), CHH hypermethylation in gene promoters is correlated with the upregulation of development-related genes (Hamid et al. 2023; Yang et al. 2025). These findings support a potential dual role of CHH methylation, although transcriptional repression remains predominant. Our results (Fig. 5 and Fig. S1) are consistent with this context-dependent behavior, in which CHH methylation may function as either a repressive or activating mark depending on the tissue type.

Pearl millet has a relatively large genome (~ 2 Gb). Consequently, its genome is characterized by extensive non-coding regions (90.99% of the genome) and many TE-derived regions (69.36% of the genome) (Yan et al. 2023). Our analysis revealed that TE regions exhibit elevated levels of DNA methylation, particularly in the CG and CHG contexts (Fig. S17). This is consistent with the roles that DNA methylation plays in TE silencing and potentially genome stability by limiting the potential of TEs to disrupt genic and regulatory regions. The methylation of TEs not only affects the expression of proximal genes but also can affect distant genes by disrupting 3D chromatin organization (Sun et al. 2022b). Furthermore, over 40% of ACRs overlapped with TE regions, particularly LTR elements, implying that TEs can also contribute to the active chromatin state. Similar observations have also been reported in maize (Liu et al. 2025). In model species such as Arabidopsis, rice, maize, and wheat, the effects of ACRs and DNA methylation on the expression of TE-associated genes appear to be broadly conserved. TEs are abundantly distributed across these genomes, and their transposition activity is predominantly repressed through epigenetic mechanisms such as DNA methylation (Cai et al. 2022; Domínguez et al. 2020). In maize, 5.6% of TE insertions in ACRs are associated with significant alterations in the expression of nearby genes (Noshay et al. 2021; Miao et al. 2024) . In rice, the TE-derived gene *PANDA* epigenetically regulates inflorescence architecture and grain size, as loss-of-function mutations lead to reduced panicle number and increased grain weight (Mao et al. 2022). In wheat, TEs are primarily silenced via sequence hypermethylation (Xie et al. 2023). Collectively, these cross-species studies, including the current analysis (Fig. 6 and Fig. S16), support a conserved role for TEs and their associated epigenetic modifications in modulating gene expression and stress response pathways across diverse plant genomes.

While both histone modifications and chromatin accessibility regulate transcription, our results reveal interesting tissue-dependent regulatory features. The highly expressed genes in YP were characterized by strong enrichment of H3K4me3. In contrast, the highly expressed genes in RO were characterized by strong ACR signals, suggesting a major contribution from chromatin accessibility. Similar tissue-dependent regulatory patterns have been reported in various crops. In rice, H3K4me3 is dynamically enriched in floral tissues and is associated with gene activation (Zhao et al. 2020). In wheat, genes in spike tissues exhibit strong H3K4me3 enrichment and chromatin accessibility, jointly driving tissue-specific expression (Lin et al. 2024). In sorghum (*Sorghum bicolor*), ACRs are highly enriched around TSS, exhibiting low DNA methylation and elevated H3K4me3 and H3K36me3 levels, suggesting that these epigenetic features collectively contribute to transcriptional activation through a coordinated regulatory mechanism (Zhou et al. 2021). The organ-specific distribution of H3K4me3 and H3K36me3 also correlates with transcriptional activation in cotton and tomato (Han et al. 2023; Julian et al. 2023). These results emphasize the potential differences in transcriptional regulatory mechanisms across tissues, while also underscoring the importance of constructing epigenetic maps across tissues for comparison.

Our integrative analysis of ACRs, histone modifications, and transcriptomic profiles across RO, YP, and NL revealed a pivotal role for WRKY TFs in root-specific regulatory programs. In particular, WRKY55, WRKY40, and WRKY50 emerged as candidate regulators due to both the significant enrichment of WRKY binding motifs and their high expression levels, specifically in RO. Moreover, the relatively low H3K4me3 levels observed at these WRKY-regulated genes suggest that chromatin accessibility, rather than histone activation marks, may play a dominant role in facilitating their expression. The presence of WRKY motifs within root-specific ACRs suggests that these TFs actively engage with dynamic *cis*-regulatory elements to modulate gene expression, consistent with the key roles of WRKY TFs in root development (Chen et al. 2017; Li et al. 2021a). WRKY TFs modulate JA signaling by activating or repressing JA-responsive genes, thereby shaping stress-induced transcriptional programs in a tissue-specific manner (Caarls et al. 2015). WRKY75 activates JA-responsive genes and enhances JA-mediated defense responses in *Arabidopsis* (Chen et al. 2021), while in tomato, SlWRKY50 promotes cold tolerance by inducing JA biosynthesis and is itself transcriptionally regulated by SlMYC2, a key JA signaling component (Wang et al. 2024).

Consistent with these findings, in pearl millet, genes associated with gained DARs in RO were significantly enriched for the JA signaling pathway, a key phytohormonal pathway involved in stress responses and root development. Our analysis points to a potential regulatory axis in which WRKY TFs bind to accessible chromatin at JA-responsive genes, orchestrating transcriptional programs that underpin root-specific developmental processes and environmental adaptation. WRKY TFs are also implicated in regulating chromatin structure, highlighting their potential role in modulating gene accessibility under stress conditions (Li et al. 2024). Further investigation into the binding dynamics of WRKY TFs and their influence on chromatin accessibility will provide deeper insight into the epigenetic regulation of JA-mediated stress responses. We identified only 980 specific ACRs in YP, which is substantially fewer than those observed in RO (5269). This discrepancy may be partially explained by the relatively low quality and complexity of the YP samples.

In conclusion, the epigenetic maps of RO and YP tissues in pearl millet generated in this study reveal a cooperative effect of DNA methylation, histone modification, and chromatin accessibility in determining tissue-specific transcription. Our findings highlight the complex and dynamic interplay among epigenetic mechanisms. In addition, they provide systematic data to facilitate the annotation of the functional non-coding genome in various pearl millet tissues.

## Materials and methods

### Plant materials and growth conditions

Seeds of *Cenchrus americanus* cultivar ‘Tifleaf 3’ were obtained from Sichuan Agricultural University and cultivated in the germplasm resource nursery of the National Engineering Research Center of JUNCAO Technology. Mature seeds were harvested, and germination was conducted as described previously (Luo et al. 2025). After germination, the seedlings were transplanted into small pots (10 cm × 15 cm) containing a cultivation substrate composed of peat and vermiculite mixed in a 3:1 ratio. After 15 days of growth, the seedlings were transferred to larger pots (20 cm × 20 cm) and maintained in a climate chamber under controlled conditions (28 °C, 14-h light/10-h dark photoperiod). After 85 days of growth, RO and YP were collected for ATAC-seq, RNA-seq, and WGBS. ChIP-seq data from RO and YP, along with multi-tissue epigenomic data from leaves, were obtained from our previous publication (Sun et al. 2023) and further analyzed in this study.

### ATAC-seq experiment and data analysis

Nuclei were extracted from *C. americanus* as described previously with some modifications (Wen et al. 2023). Approximately 0.05–1 g of RO and YP tissue was collected and transferred to a pre-chilled Petri dish placed on ice. The tissues were finely chopped using a sterile single-edged razor blade in 1 mL of ice-cold lysis buffer to ensure thorough homogenization. The homogenized sample was transferred to a 2-mL centrifuge tube, rinsed with an additional 1 mL of lysis buffer to recover residual material, and incubated at 4 °C with gentle rotation for 30 min. After incubation, the lysate was filtered through a 40-μm cell strainer and gently layered onto 2 mL of pre-chilled sucrose cushion buffer (2.4 M sucrose, 20 mM Tris–HCl pH 8.0, 2 mM MgCl_2_, 2 mM EDTA, 15 mM 2-ME, and 0.2% Triton X-100) in a 15-mL centrifuge tube. Nuclei were pelleted by centrifugation at 2200 *g* for 20 min at 4 °C. The resulting pellet was washed with 1 mL of nuclei wash buffer (10 mM Tris–HCl pH 8.0, 5 mM MgCl_2_), centrifuged again at 1200 *g* for 5 min at 4 °C, and resuspended for downstream analysis. The isolated nuclei were stained with DAPI (CAS 47165–04-8; LABLEAD, China) in the dark for 5 min and examined under a fluorescence microscope (BX3-CBH; OLYMPUS, Japan) to assess nuclear integrity. Intact nuclei were incubated with Tn5 transposase for simultaneous DNA fragmentation and adapter integration. DNA purification, library amplification, and size selection were carried out using a TruePrep ATAC-seq Kit V2 for Illumina (TD202, Vazyme Biotech) according to the manufacturer’s instructions. The final libraries were sent to Annoroad Gene Technology (Beijing, China) for paired-end sequencing (2 × 150 bp) on the MGI T7 platform (MGI Tech, China).

Raw reads were trimmed with fastp (version 0.23.4), and reads shorter than 50 bp were discarded (Chen et al. 2018). Clean reads were aligned to the pearl millet PI537069 reference genome (Yan et al. 2023) using Bowtie2 (version 2.5.1) with the parameters “–local –very-sensitive –no-mixed –no-discordant -X 2000” to allow for precise alignment (Langmead and Salzberg 2012). The aligned reads were sorted using SAMtools (version 1.18), and low-quality alignments with mapping quality (MAPQ) < 20 were removed. PCR duplicates were then identified and removed using Sambamba markdup (version 1.0.0) with default settings to retain only non-redundant, uniquely mapped fragments for downstream ATAC-seq analysis (Tarasov et al. 2015). Chromatin accessibility peaks were called using MACS2 (version 2.2.9.1) with the parameters “–nomodel –shift -75 –extsize 150 –qvalue 0.01 –call-summits”. A *q*-value threshold of 0.05 was used to control the false discovery rate, ensuring high-confidence peak identification (Zhang et al. 2008). According to the definition at ENCODE (https://www.encodeproject.org/data-standards/terms/#enrichment), the TSS enrichment score was computed using ATACseqQC (version 1.15.8) (Ou et al. 2018). The R package DiffBind (version 3.12.0) was utilized to identify potential DARs (Stark and Brown 2011). Differential binding region analysis was performed using the dba.analyze function in DESeq2. Differential accessibility was assessed by comparing normalized read counts across conditions, and statistically significant DARs were defined as those with a *P*-value < 0.05.

### RNA-seq library preparation and data analysis

RO and YP were harvested and placed in 2-mL centrifuge tubes containing 4-mm stainless steel beads. Approximately 0.1 g of each sample was homogenized in liquid nitrogen using a high-throughput tissue grinder (Shanghai Jingxin Industrial Development, China) at 50 Hz for 90 s. The samples were sent to Novogene (China) on dry ice for RNA extraction and library preparation. Standard protocols were used for mRNA enrichment, cDNA synthesis, and library construction. RNA-seq was conducted with two biological replicates per tissue, generating approximately 6 Gb of sequencing data per sample. Genome sequence and annotation files were downloaded from the EGDB website (https://engrass.juncaodb.cn/). RNA-seq was performed as previously described (Luo et al. 2025). Differentially expressed genes were detected using the edgeR package; significant DEGs were defined as genes with log_2_|fold change|> 1 and adjusted *p*-value ≤ 0.05. Gene functions were annotated using eggNOG (Huerta-Cepas et al. 2019), and GO and KEGG enrichment analyses were performed using clusterProfiler (Yu et al. 2012).

### WGBS and data analysis

RO and YP were ground into a fine powder in liquid nitrogen using a high-throughput tissue grinder. Genomic DNA was extracted from the samples using a Hi-DNAsecure Plant Kit (DP350; TIANGEN Biotech, China) following the manufacturer’s instructions. For each sample, 1 μg of high-quality genomic DNA was used for WGBS, which was conducted by Annoroad Gene Technology (China). DNA fragmentation, end repair, A-tailing, and ligation with methylated adapters were performed, followed by fragment selection using 2% agarose gel electrophoresis. Bisulfite conversion was carried out using the EZ DNA Methylation-Gold™ Kit (D5006; Zymo Research, USA) according to the manufacturer’s protocol. After PCR amplification and purification, the libraries were sequenced on the MGI T7 platform (MGI Tech, China) using paired-end reads (2 × 150 bp).

Raw reads were filtered using fastp (version 0.23.4) with the parameters “-W 5 -M 20 -5 -3 -l 50”, and the clean reads were mapped to the pearl millet genome using Bismark (version 0.24.2) with default parameters (Krueger and Andrews 2011). After removing PCR duplicates from the aligned reads, cytosine methylation information was extracted for each site using Bismark Methylation Extractor. Only sites covered by more than three reads were retained for further evaluation. The methylation profiles in genic regions and other regions of interest were calculated and visualized in R (version 4.4.2). DMRs were identified by DMRcaller (v1.26.0) to identify 100-bp bins containing more than 5 cytosines, with only 10 cytosines covered for each cytosine used to detect DMRs. DMRs were detected with a *p*-value < 0.05 and methylation difference thresholds of 0.4%, 0.2%, and 0.1% for CG, CHG, and CHH, respectively. Differentially methylated genes were defined as genes with DMRs in their promoter regions (2 kb upstream of the TSS).

## Supplementary Information

Below is the link to the electronic supplementary material.

Supplementary file1 (DOCX 3262 KB)

Supplementary file2 (XLSX 13 KB)

## Data Availability

The raw sequence data in this study were deposited in the China National GeneBank Database (CNGBdb) at https://ngdc.cncb.ac.cn/bioproject/browse/PRJCA037501 and on the EGDB website (https://engrass.juncaodb.cn/download.html).
