# Supplementary material for: Epigenetic maps of pearl millet reveal a prominent role for CHH methylation in regulating tissue-specific gene expression
Source: aBIOTECH. 2025 Aug 26;6(3):394–410. doi: 10.1007/s42994-025-00243-2 (PMC12454808; doi:10.1007/s42994-025-00243-2)
Supplement: Supplementary file 1 — Supplementary file1 (DOCX 3262 KB) [file 42994_2025_243_MOESM1_ESM.docx]

**Supporting information to**

**Epigenetic maps of pearl millet reveal a prominent regulatory role of CHH methylation**

Lin Luo^1#, 3^, Qi Qu^1#^, Mengxue Cao^1^, Yihui Zhang^1^, Yuanchang Sun^1^, Fei Mao^2^, Jiaming Chen^2^, Yilin Zhu^1^, Yaorou Yang^1^, HuachengLiu^1^, Chunxiao Li^1^, Dongmei Lin^2^, Guodong Lu^1^, Zhanxi Lin^2^, Fangjie Zhu^1*^, Jiajing Xiao^1*^

^1^College of Life Science, National Engineering Research Center of JUNCAO, Fujian Provincial Key Laboratory of Haixia Applied Plant Systems Biology, Haixia Institute of Science and Technology, Fujian Agriculture and Forestry University, Fuzhou 350002, China

^2^Juncao Science and Ecology College, Fujian Agriculture and Forestry University, Fuzhou 350002, China

^3^Life Sciences Institute, Zhejiang University, Hangzhou 310058 China

Lin Luo and Qu Qi contributed equally to this study.

*Correspondence:

[jjxiao@fafu.edu.cn](mailto:jjxiao@fafu.edu.cn%20) (Jiajing Xiao),

[fjzhu@fafu.edu.cn](mailto:fjzhu@fafu.edu.cn) (Fangjie Zhu)

**
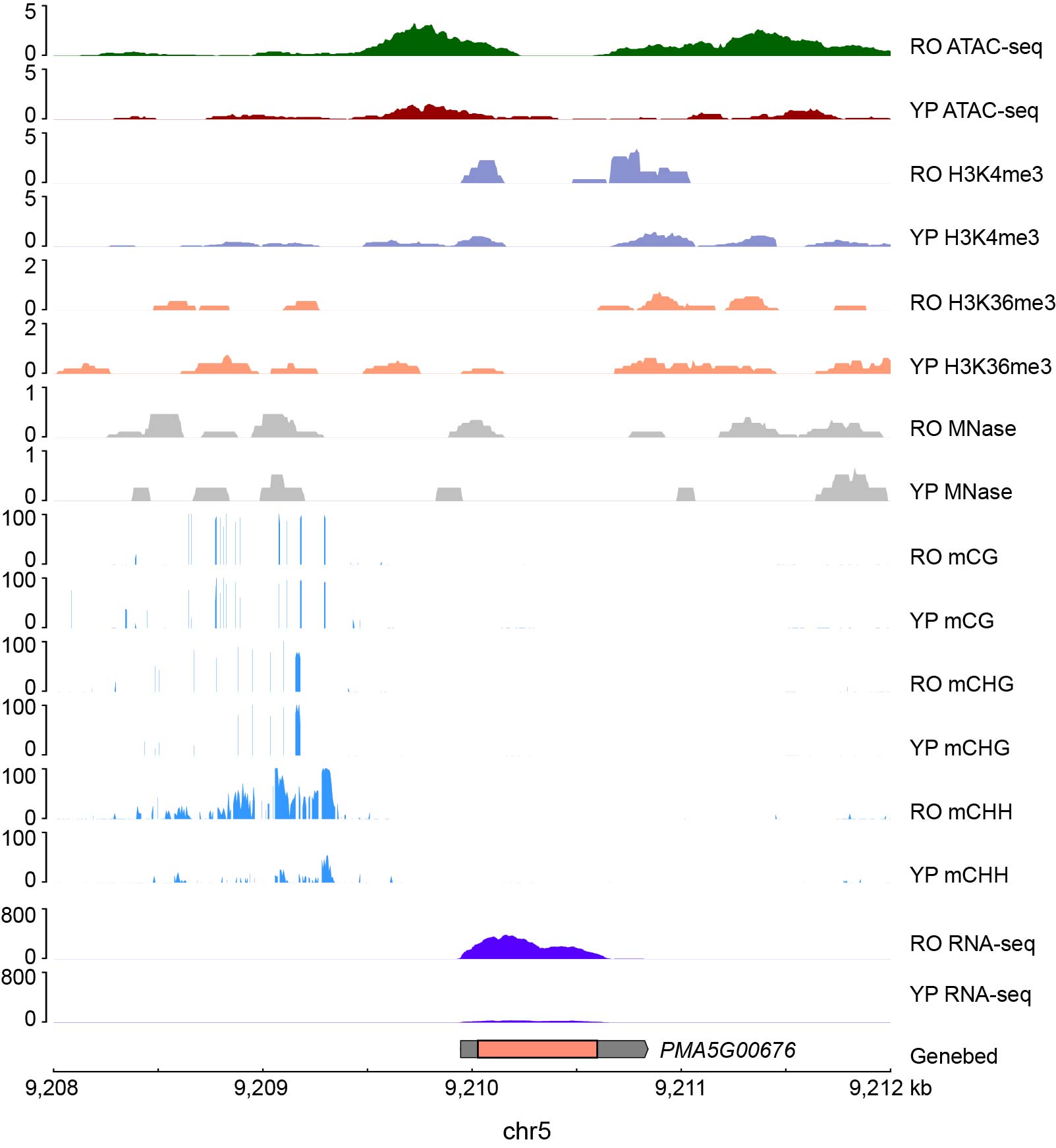
**

**Fig. S1.** Integrated epigenomic and transcriptomic landscape illustrating elevated CHH methylation in the promoter region of *PMA5G00676* in root (RO). Tracks from top to bottom display chromatin accessibility profiles (ATAC-seq) in RO (green) and young panicle (YP, red), followed by histone modification enrichment (H3K4me3 and H3K36me3) in both tissues. Nucleosome positioning was assessed using MNase-seq in RO and YP. DNA methylation patterns in CG, CHG, and CHH sequence contexts are shown for each tissue, with methylation levels indicated by vertical bars. Transcript abundance based on RNA-seq is presented for RO and YP. The gene model of *PMA5G00676* is shown at the bottom, with exons represented as boxes and introns as connecting lines. This integrative analysis reveals the chromatin features and root-specific epigenetic regulation associated with tissue-dependent expression of *PMA5G00676*.


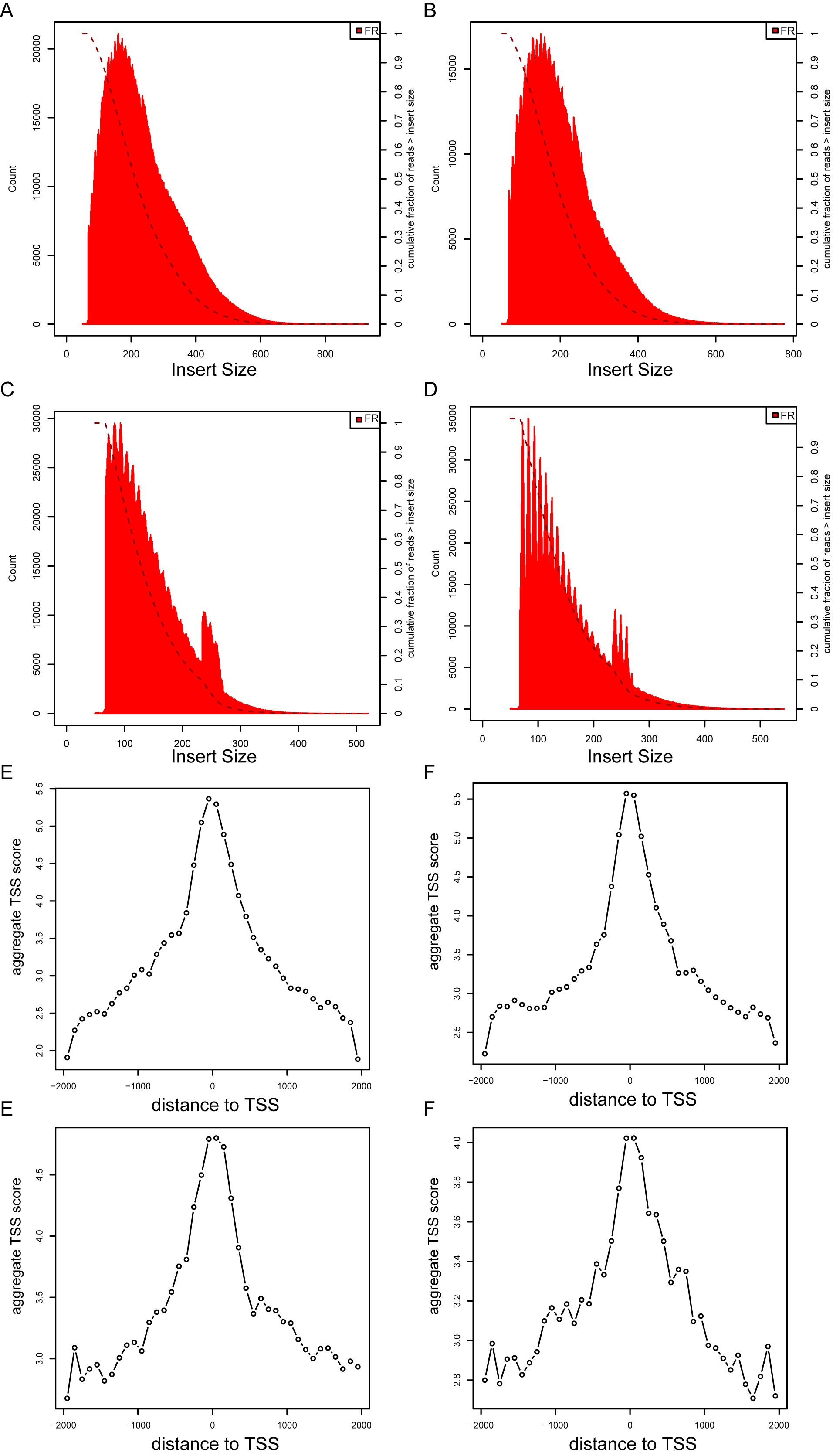


Fig. S2. Quality assessment of ATAC-seq libraries in root (RO) and young panicle (YP) tissues. (A–D) Insert size distribution of ATAC-seq fragments for RO_1 (A), RO_2 (B), YP_1 (C), and YP_2 (D), displaying the characteristic periodicity corresponding to nucleosome-free fragments and mono-, di-, and tri-nucleosome-bound fragments. (E–H) Transcription start site (TSS) enrichment profiles for RO_1 (E), RO_2 (F), YP_1 (G), and YP_2 (H), demonstrating robust signal enrichment around TSSs, indicative of high-quality chromatin accessibility data.


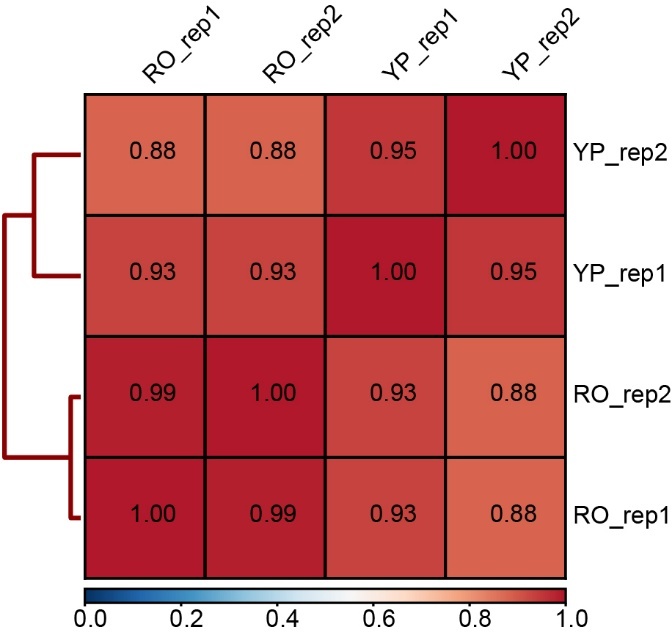


Fig. S3. Pearson correlation analysis of ATAC-seq signal profiles reveals high reproducibility in RO and YP tissues. Pairwise Pearson correlation coefficients were computed using deepTools, based on genome-wide ATAC-seq signal tracks normalized to BPM (bins per million mapped reads). The resulting heatmaps illustrate high reproducibility between biological replicates within each tissue, confirming the consistency and reliability of the chromatin accessibility data.


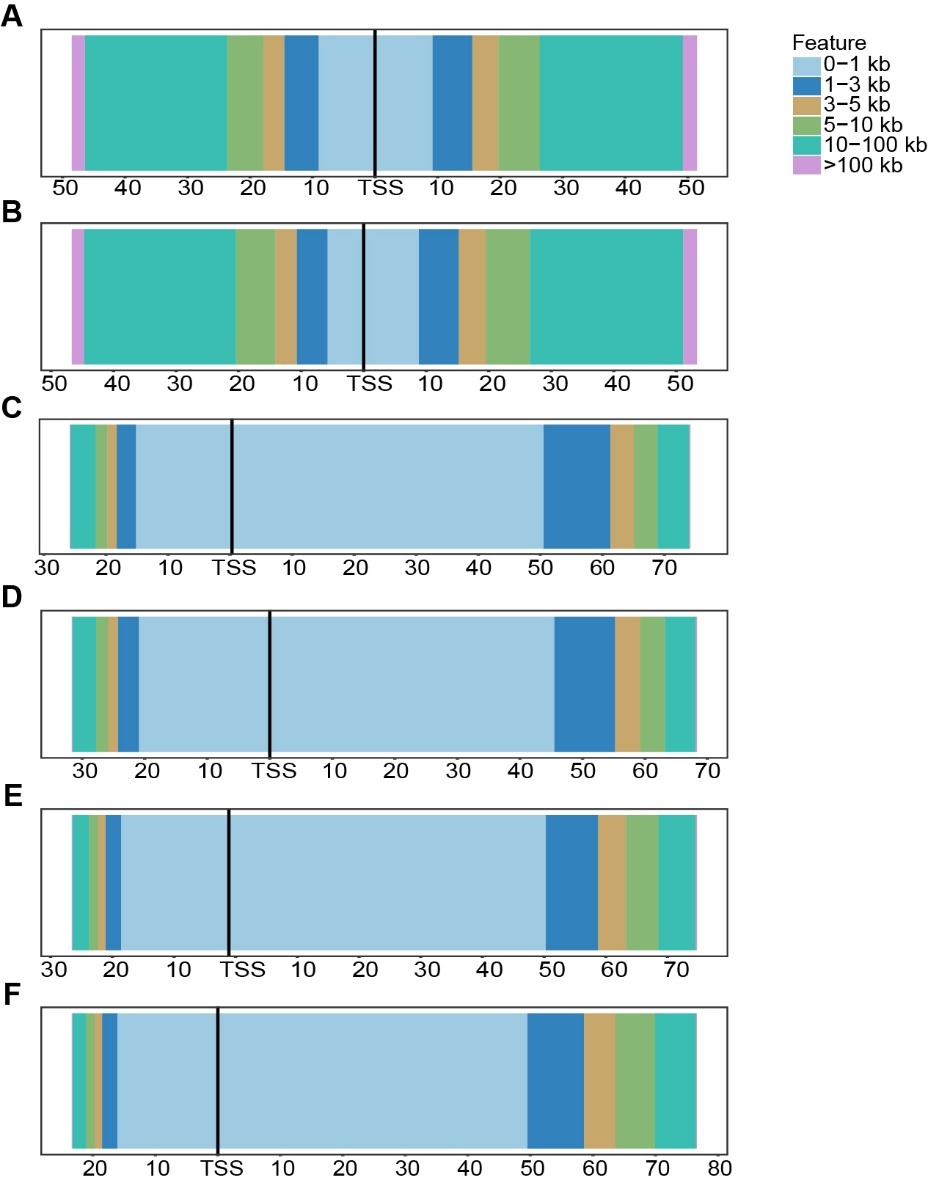


Fig. S4. Genomic distribution of chromatin features relative to transcription start sites (TSSs) in root (RO) and young panicle (YP) tissues. (A–B) Distribution of ATAC-seq peaks in RO (A) and YP (B). (C–D) Distribution of H3K4me3-enriched regions in RO (C) and YP (D). (E–F) Distribution of H3K36me3-enriched regions in RO (E) and YP (F). Each panel displays the proportion of features located at varying distances from the nearest TSS, binned into six categories: 0–1 kb, 1–3 kb, 3–5 kb, 5–10 kb, 10–100 kb, and >100 kb.


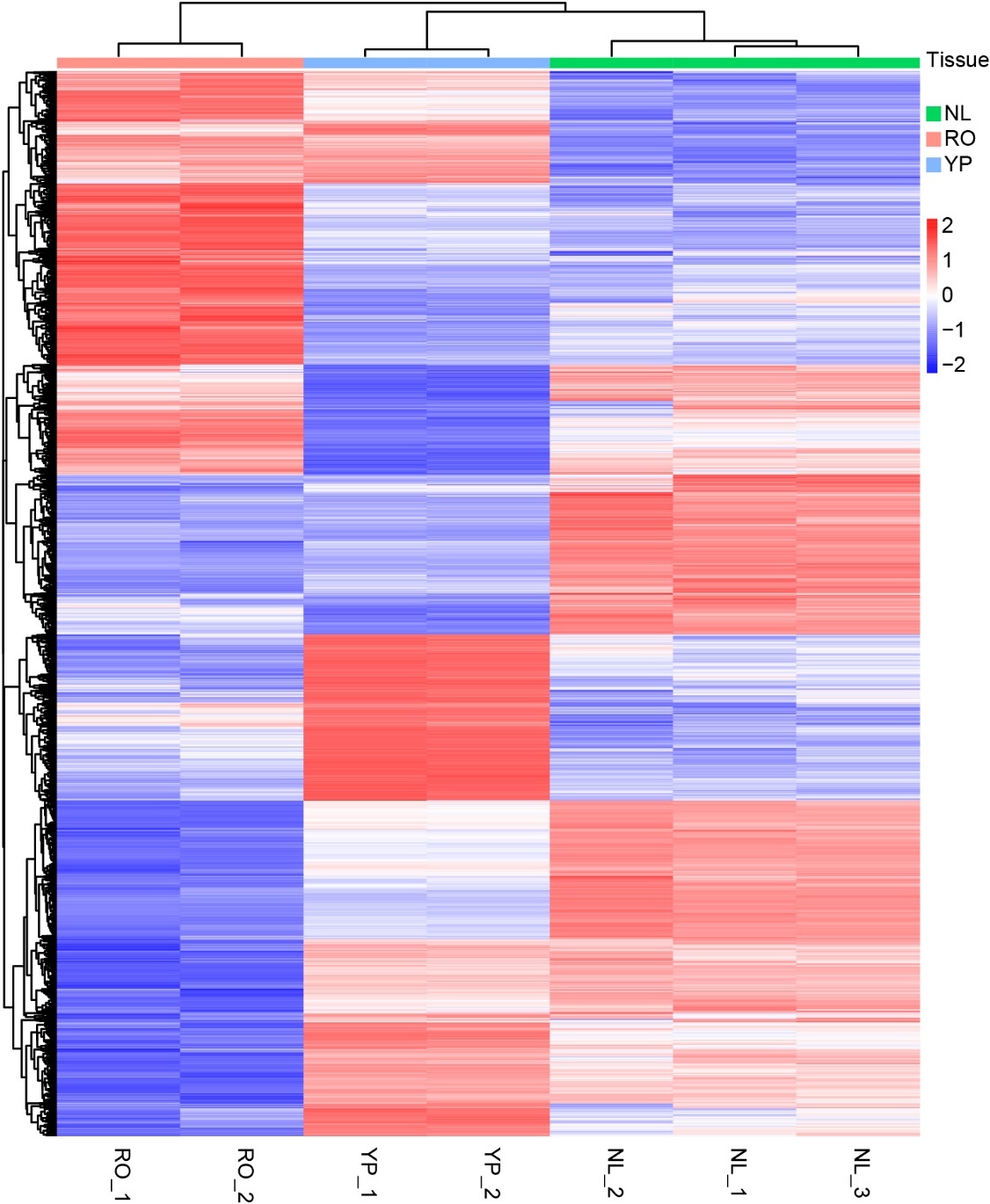


Fig. S5. Heatmap of differentially expressed genes (DEGs) across RO, YP, and new leaf (NL) tissues. Gene expression values, based on normalized FPKM (Fragments Per Kilobase of transcript per Million mapped reads), were log₂(FPKM + 1)-transformed and subsequently subjected to row-wise z-score normalization to emphasize relative expression variation across samples. Hierarchical clustering of both genes (rows) and samples (columns) reveals distinct tissue-specific expression patterns. Red and blue represent relatively high and low expression levels, respectively. This analysis highlights transcriptomic divergence among the three tissue types.


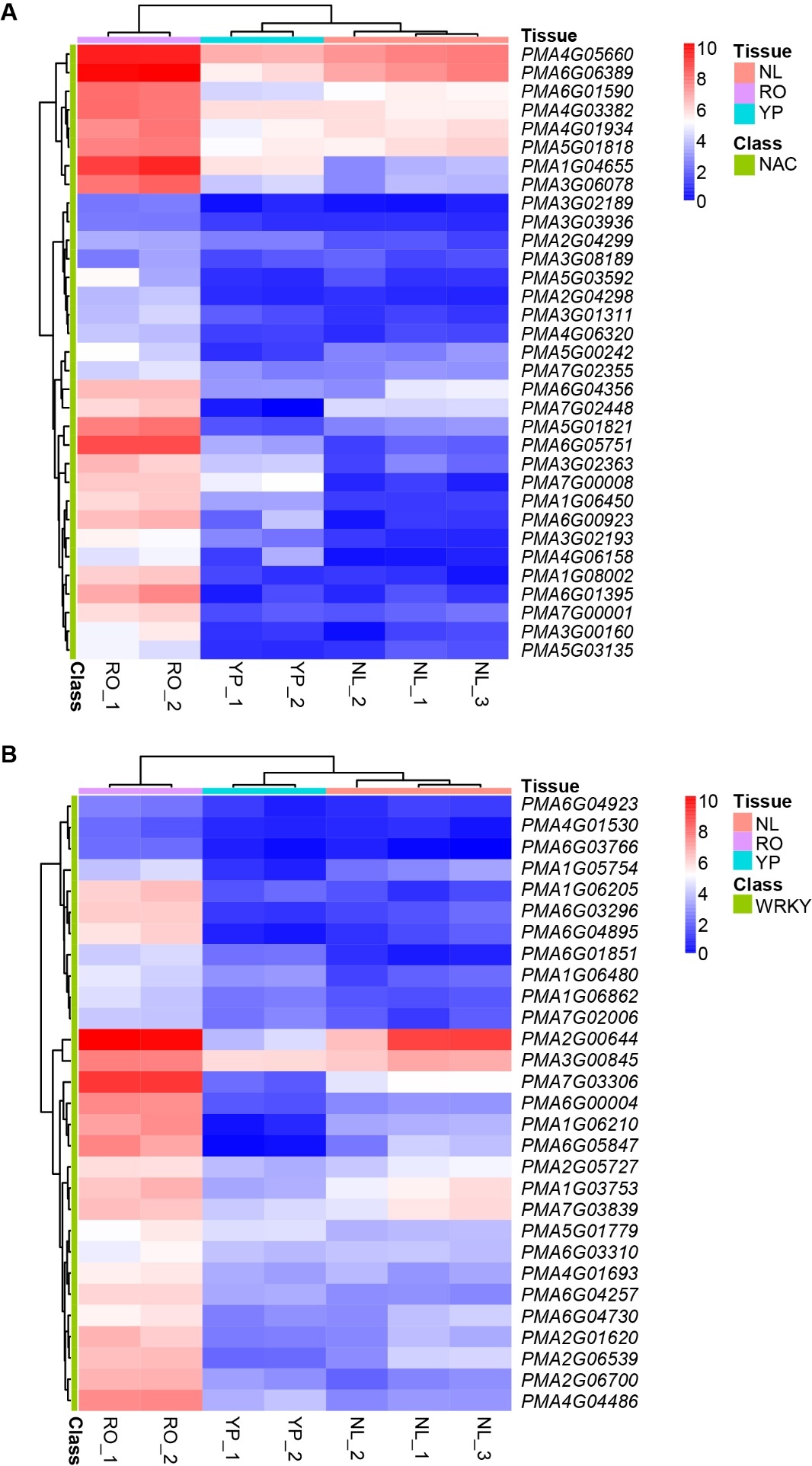


Fig. S6. Heatmaps of differentially expressed NAC (A) and WRKY (B) transcription factor genes across RO, YP, and NL tissues. Gene expression values were transformed using log₂(FPKM + 1) and visualized by hierarchical clustering of both genes (rows) and samples (columns). Tissue types are indicated by color annotations, and transcription factor classes (NAC and WRKY) are labeled alongside each heatmap. Red and blue indicate relatively high and low expression levels, respectively. These results highlight distinct tissue-specific expression patterns of NAC and WRKY transcription factor families.


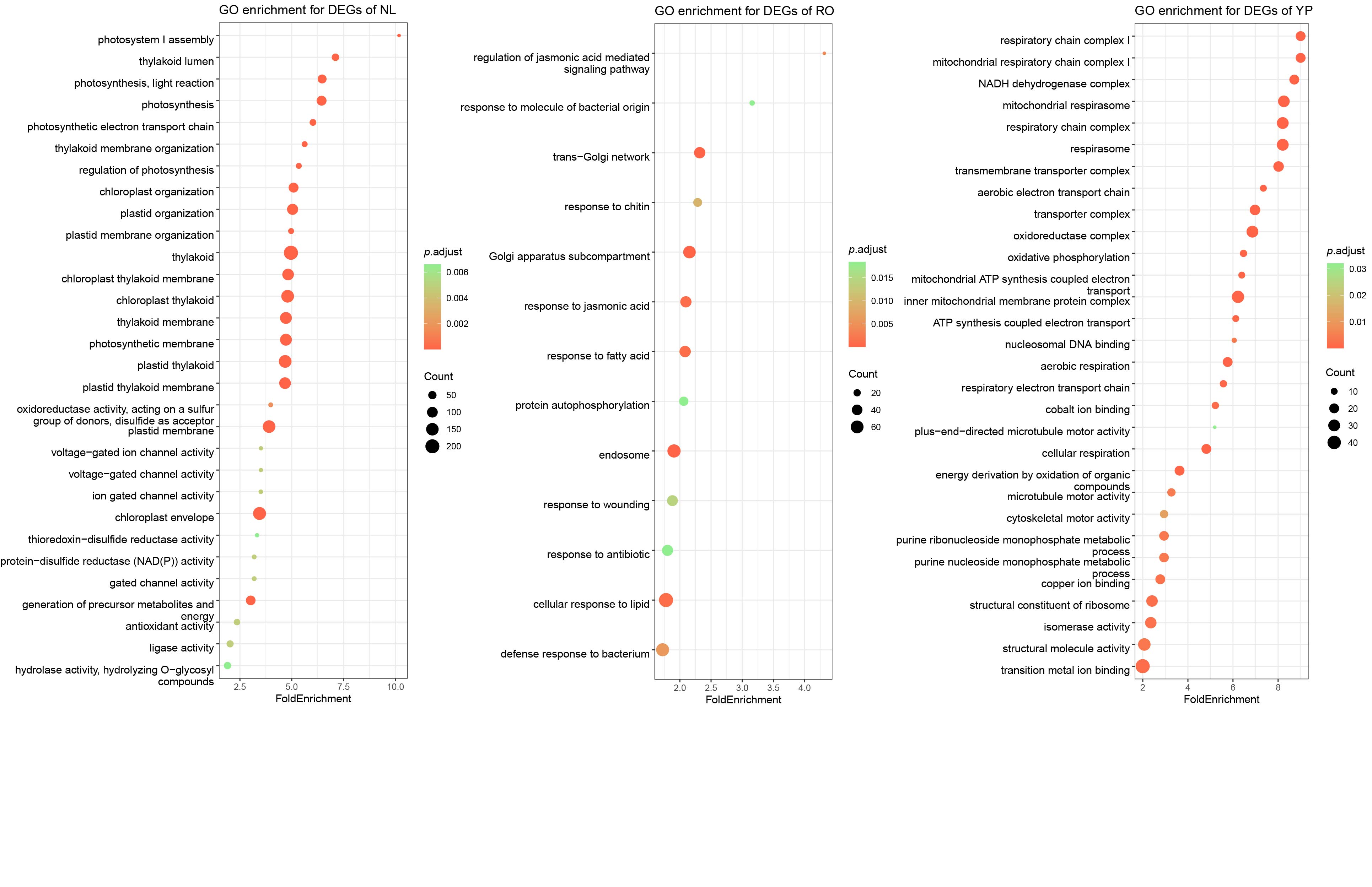


Fig. S7. Bubble plots of Gene Ontology (GO) enrichment for differentially expressed genes (DEGs) in RO, NL, and YP tissues. GO enrichment analyses were performed separately for RO (root), NL (leaf), and YP (young panicle) using DEGs identified from each tissue type. The y-axis represents enriched GO terms, and the x-axis shows the fold enrichment. Dot size indicates the number of DEGs associated with each term (Gene Count), and dot color represents the adjusted p-value (*p*.adjust), with more significant terms shown in red. Only the top GO terms (based on significance and gene count) are displayed for clarity. The GO terms displayed in the enrichment analysis represent the top 10 most significant terms within each Gene Ontology (GO) category—Biological Process (BP), Molecular Function (MF), and Cellular Component (CC)—selected based on ascending order of the adjusted *p*-value (*p*. adjust).


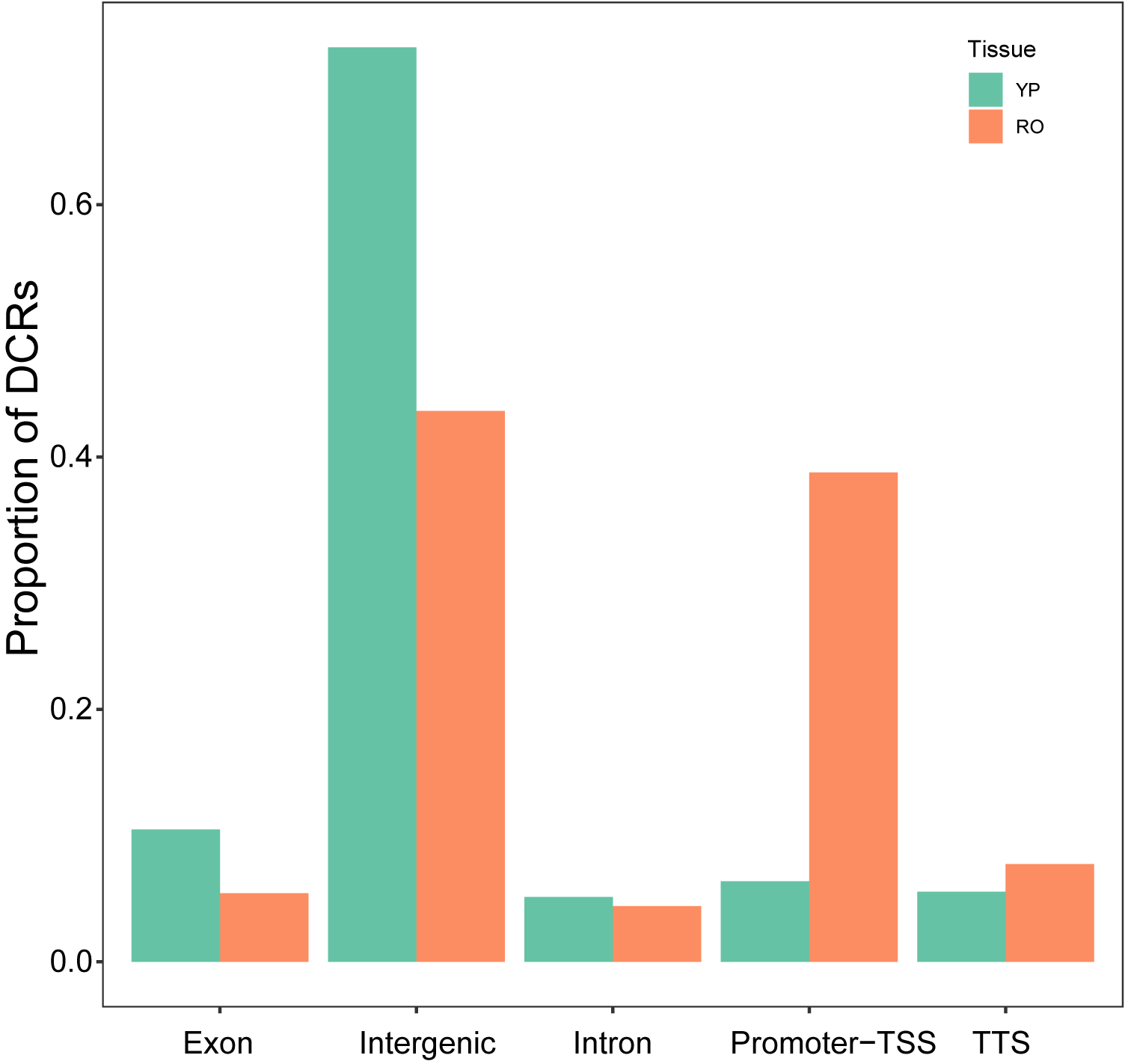


Fig. S8. Genomic annotation of tissue-specific accessible chromatin regions (ACRs) in root and panicle. Bar plot showing the proportion of differentially accessible chromatin regions (DCRs) assigned to major genomic features in root (RO) and panicle (YP) tissues. Genomic features include exons, intergenic regions, introns, promoter regions (within 2 kb upstream of TSS), and transcription termination sites (TTS). Root-specific DCRs are predominantly enriched in promoter-TSS and intergenic regions, while panicle-specific DCRs are more heavily biased toward distal intergenic regions, suggesting differences in the regulatory architecture between tissues.


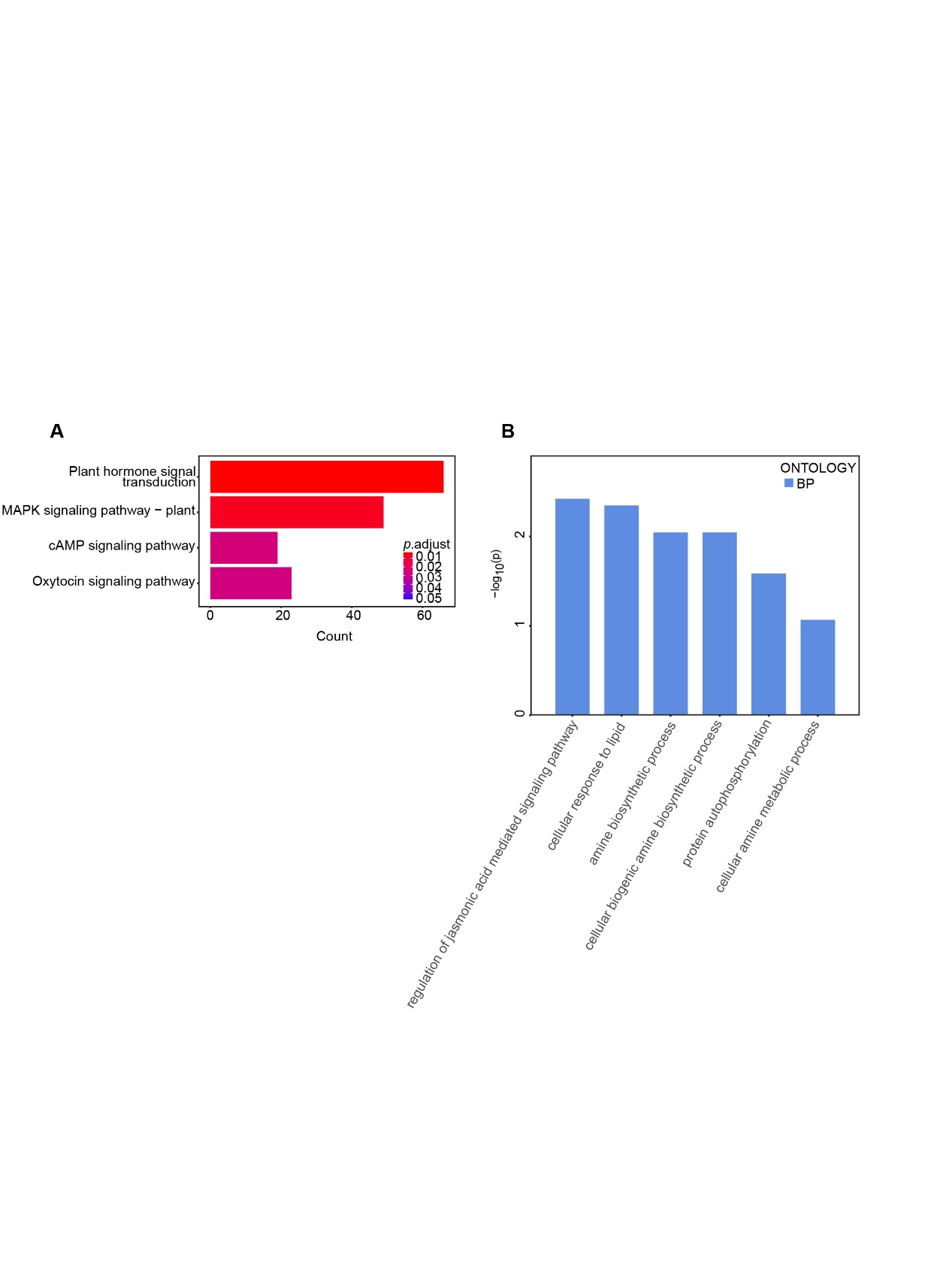


Fig. S9. Enriched GO and KEGG terms for genes associated with hormone signaling and metabolic reprogramming linked to gained differentially accessible regions (DARs) in RO tissue. (A) KEGG pathway enrichment analysis reveals significant enrichment of signaling pathways, including plant hormone signal transduction and MAPK signaling. Bar length indicates the number of associated genes, while bar color represents adjusted *p*-values. (B) Gene Ontology (GO) biological process (BP) enrichment analysis of the same gene set highlights processes such as regulation of jasmonic acid-mediated signaling, cellular response to lipid, and amine biosynthetic pathways. Bar height reflects –log₁₀(*p*) values.


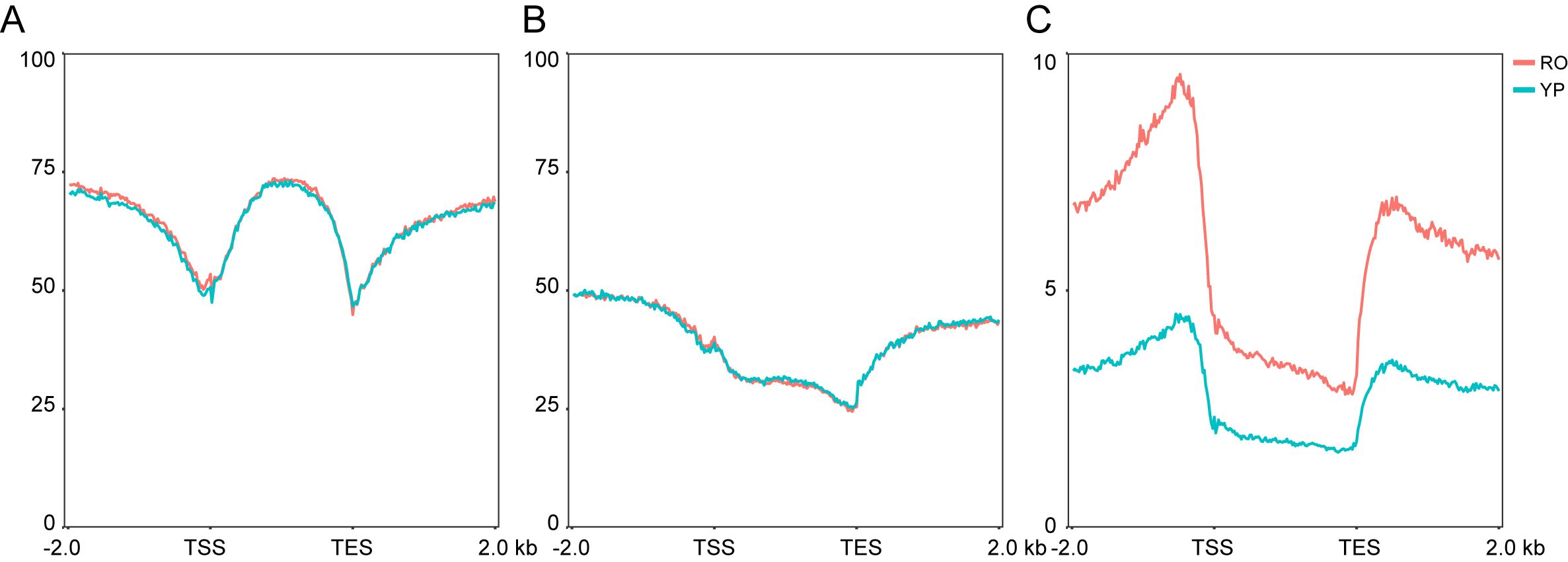


Fig. S10. DNA methylation levels across gene regions in RO (red) and YP (blue) tissues of pearl millet. Methylation profiles are shown in three sequence contexts: CG (A), CHG (B), and CHH (C). The x-axis represents regions spanning 2 kb upstream of the transcription start site (TSS), the gene body (from TSS to transcription end site, TES), and 2 kb downstream of TES. The y-axis indicates average methylation percentage. CG and CHG methylation exhibit typical depletion at TSS and TES sites with moderate differences between tissues, while CHH methylation is substantially higher in RO, particularly in flanking and gene body regions, suggesting tissue-specific epigenetic regulation.


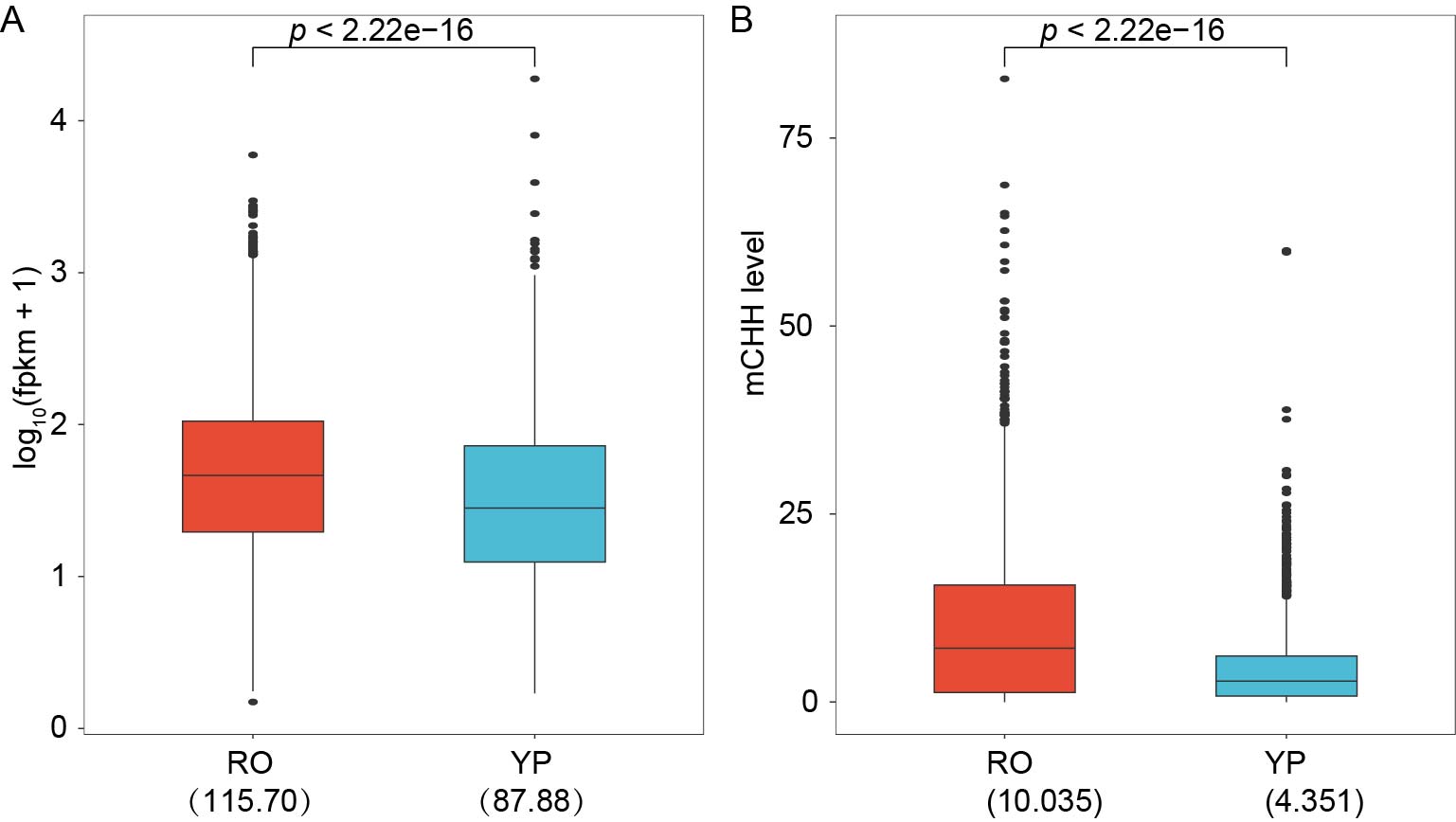


Fig. S11. Comparison of gene expression and CHH methylation levels in RO and YP tissues. (A) Boxplot showing significantly higher expression levels (log_₁₀_(FPKM + 1)) of tissue-specific genes in RO (red, mean = 115.70) than YP (blue, mean = 87.88) (*t*-test, *p* < 2.22e–16). (B) Boxplot comparing CHH methylation levels in promoter regions of tissue-specific genes in RO and YP. RO shows significantly higher promoter CHH methylation than YP (*t*-test, *p* < 2.22e–16). Median values are shown below each group in parentheses.


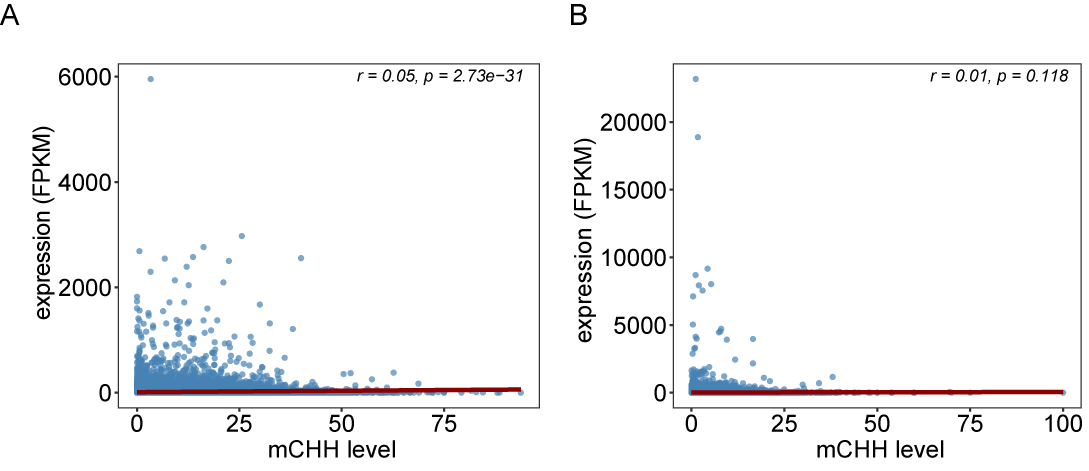


Fig. S12. Pearson correlation between CHH methylation level in promoter regions and gene expression in RO(A) and YP(B). The Pearson's correlation for RO is 0.053 with *p* <2e-16, and the Pearson's correlation for YP is 0.0072 with *p* = 0.1.


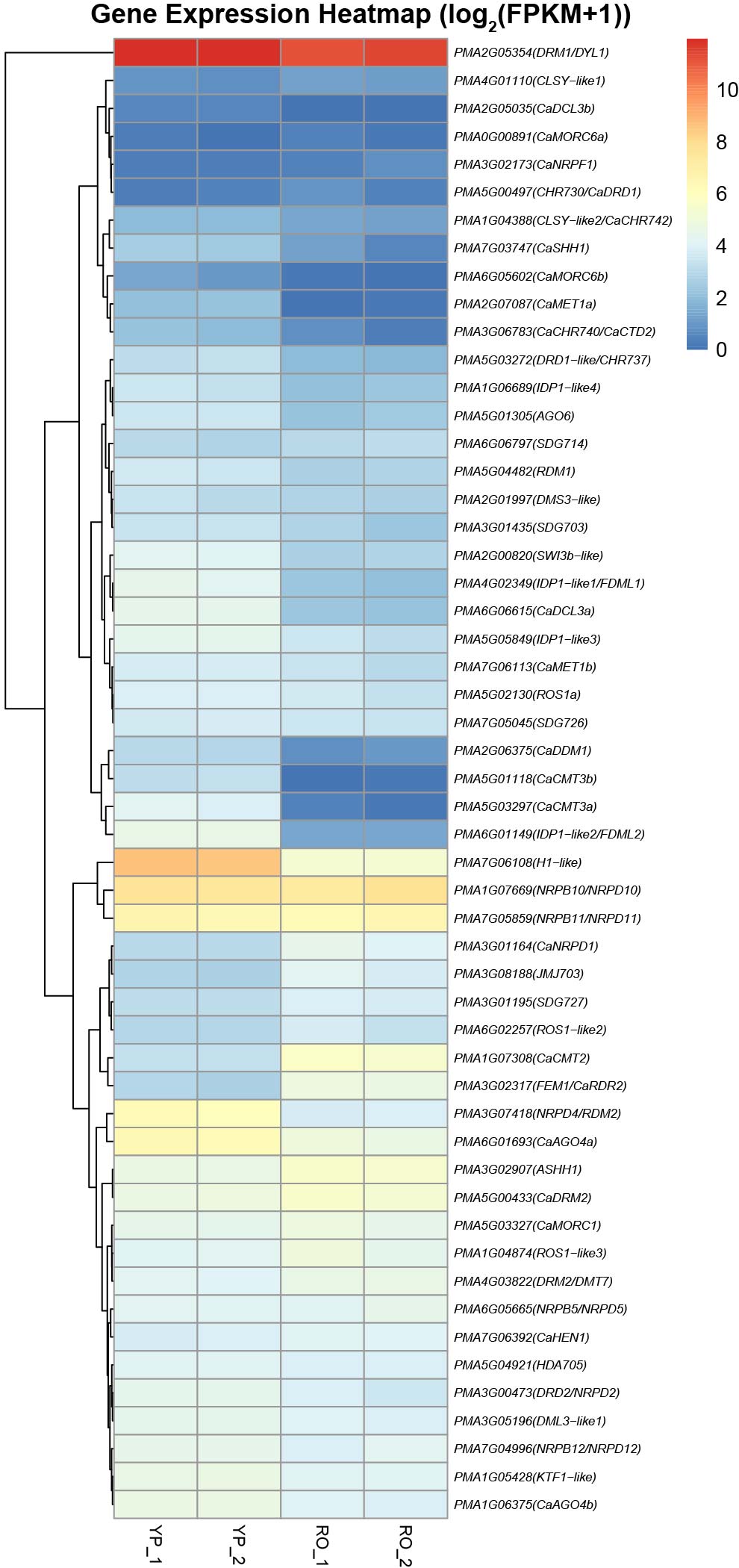


Fig. S13. Expression heatmap of putative RdDM pathway homologs in pearl millet across RO and YP tissues. The heatmap shows the expression levels (log₂(FPKM + 1)) of homologous genes involved in RNA-directed DNA methylation (RdDM) pathways, identified in the *Cenchrus americanus* genome based on *Arabidopsis thaliana* and rice orthologs. Samples include root (RO_1, RO_2) and young panicle (YP_1, YP_2).


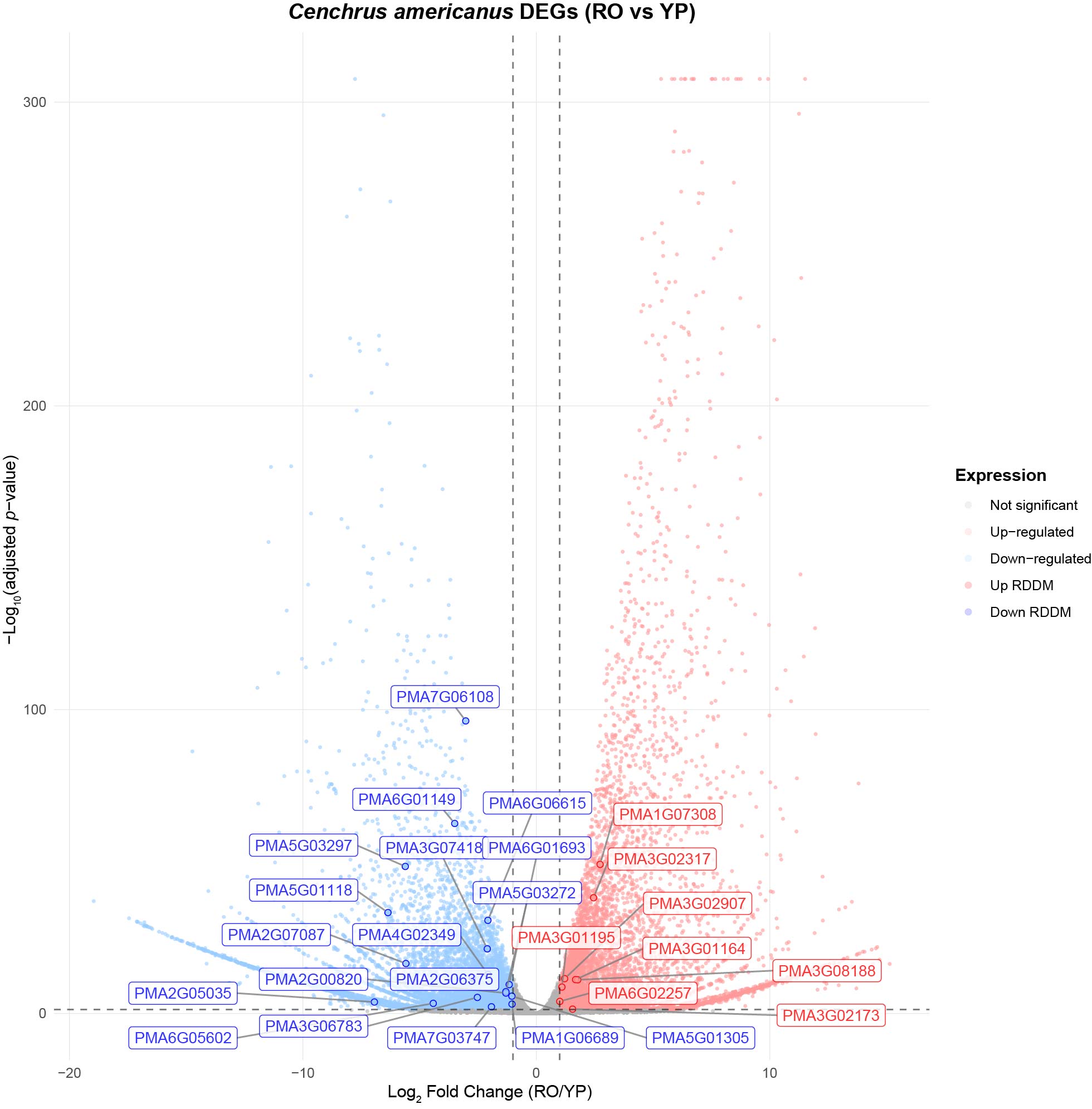


Fig. S14. Volcano plot showing differential expression of RdDM pathway-related genes between RO and YP tissues in *C. americanus*. Differentially expressed genes (DEGs) between young panicle (YP) and root (RO) were visualized using a volcano plot. The x-axis represents the log₂ fold change (YP/RO), and the y-axis shows the –log₁₀ adjusted *p*-value. Genes significantly upregulated in YP are shown in red, and those upregulated in RO are in blue. RdDM pathway-related genes are highlighted and labeled.


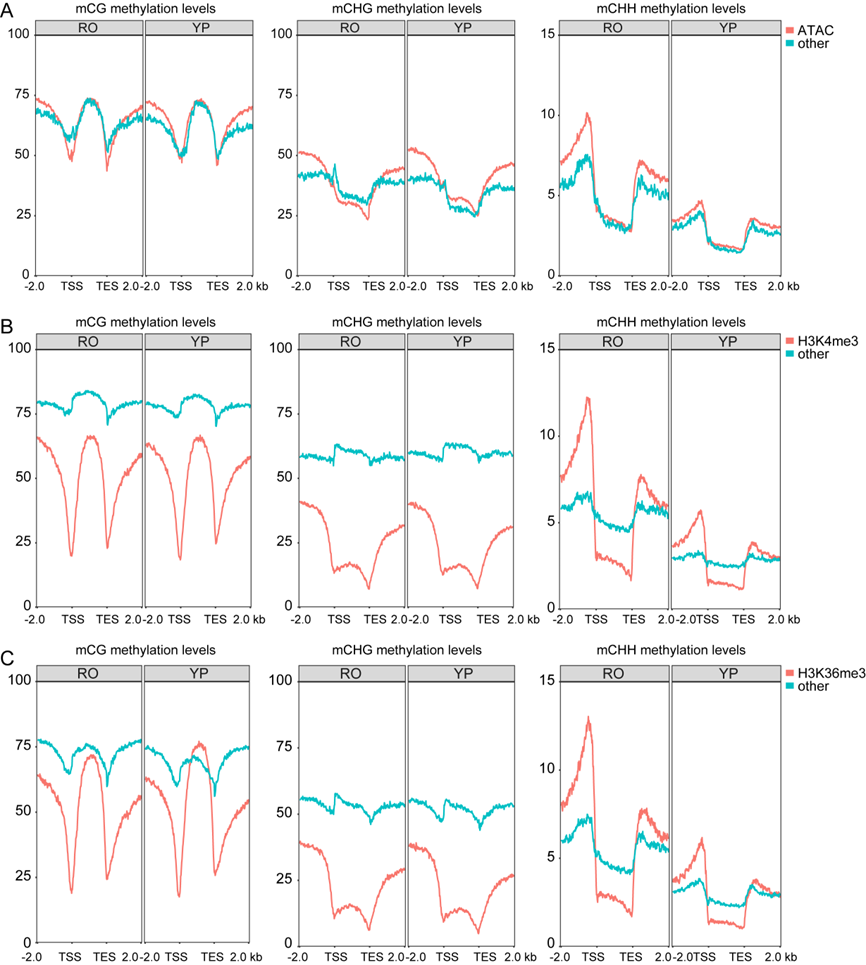


Fig. S15. DNA methylation profiles across genomic regions marked by chromatin accessibility or histone modifications in RO (red) and YP (blue) tissues of pearl millet. (A) Methylation levels across genes associated with ATAC-seq peaks (open chromatin) versus other genes in CG, CHG, and CHH sequence contexts. (B) Methylation levels across genes marked by H3K4me3 versus other genes in the three methylation contexts. (C) Methylation levels across genes marked by H3K36me3 versus other genes. For each panel, the x-axis represents the region spanning 2 kb upstream of the TSS, the gene body (TSS to TES), and 2 kb downstream of TES. The y-axis indicates average DNA methylation percentage. Genes associated with ATAC, H3K4me3, or H3K36me3 features exhibit distinct methylation profiles compared to other genes, particularly in the CHH context, suggesting coordinated regulation between chromatin features and DNA methylation in a tissue-specific manner.
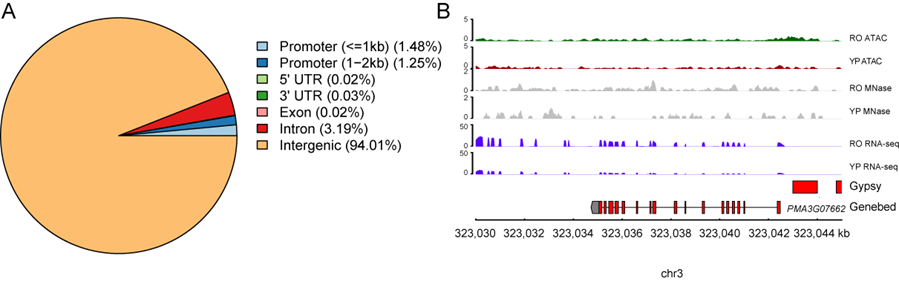


Fig. S16. Distribution and example of Gypsy-associated accessible chromatin regions (ACRs). (A) Genomic distribution of Gypsy-associated ACRs. (B) Genome browser view of a representative Gypsy-associated ACR located near the gene *PMA3G07662* on chromosome 3. Tracks show chromatin accessibility profiles (ATAC-seq), nucleosome positioning (MNase-seq), and gene expression (RNA-seq) in root (RO) and panicle (YP) tissues.


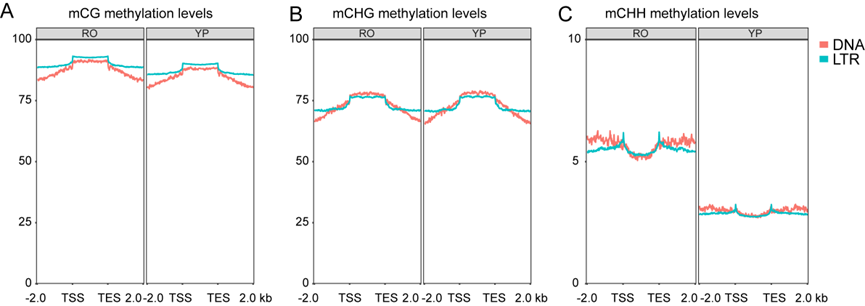


Fig. S17. DNA methylation profiles across DNA transposons (red) and LTR retrotransposons (blue) in RO and YP tissues of pearl millet. Methylation levels are shown in CG (A), CHG (B), and CHH (C) contexts for 2 kb flanking regions and the internal body (from “start” to “end”) of each transposon. Overall, both DNA and LTR transposons exhibit high levels of CG and CHG methylation, particularly within the transposon body, with LTR elements showing slightly higher CG methylation than DNA transposons. CHH methylation levels are comparatively lower but remain above background, with minimal differences between DNA and LTR elements. These patterns suggest that transposon silencing in both tissues is maintained primarily through CG and CHG methylation, with limited CHH contribution.


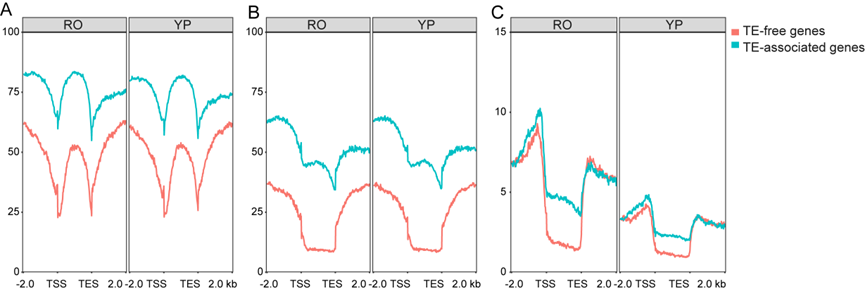


Fig.S18. DNA methylation levels across TE-associated genes (blue line) and TE-free genes (red line) in RO and YP tissues of pearl millet. Methylation levels are shown in CG (A), CHG (B), and CHH (C) contexts for 2 kb flanking regions and the internal body (from “start” to “end”) of each gene.
